# Co-Designing and Evaluating a 1-Day Quality Improvement Workshop for Medical Students and Resident Physicians: Tutorial on Applying Kern’s Curriculum Development Framework

**DOI:** 10.2196/83657

**Published:** 2026-06-17

**Authors:** Amanda Ling Jie Yee, Mariam Idrissi, Sam Sherratt-Mayhew, Charles Page, Maiar Elhariry, Punith Kempegowda

**Affiliations:** 1 Birmingham Medical School, University of Birmingham, Birmingham, United Kingdom; 2 College of Medicine and Health, Applied Health Sciences, School of Health Sciences, University of Birmingham, Birmingham, United Kingdom; 3 Sandwell and West Birmingham NHS Foundation Trust, Birmingham, United Kingdom; 4 Department of Diabetes and Endocrinology, University Hospitals Birmingham NHS Foundation Trust, Birmingham, United Kingdom; 5 Murray Learning Centre, Edgbaston, Birmingham, B15 2TT, United Kingdom, +447721930777

**Keywords:** quality improvement, medical education, curriculum development, experiential learning, mixed methods study

## Abstract

**Background:**

Despite the importance of quality improvement in advancing patient care and safety, there is limited literature describing structured, practical, and co-designed quality improvement education.

**Objective:**

This study aimed to (1) describe how learner co-design was operationalized within Kern’s 6-step curriculum development framework to develop a quality improvement workshop for medical students and resident physicians, (2) evaluate preworkshop and postworkshop changes in learners’ self-reported understanding of and confidence in quality improvement, and (3) explore participants’ attitudes toward quality improvement and their perceptions of the workshop’s relevance to future practice.

**Methods:**

Using Kern’s 6-step curriculum development model, informed by Kolb’s Experiential Learning Theory, we co-designed a 1-day quality improvement workshop with medical students and resident physicians. To address objective 1, the workshop development process was guided by a literature review and a targeted needs assessment. To address objective 2, we used a mixed methods pre-post educational evaluation design. The workshop incorporated expert-led lectures, small-group project design exercises, and peer presentations addressing audit methodology, ethical considerations, and practical implementation. Preworkshop and postworkshop surveys assessed changes in participants’ self-reported understanding of quality improvement concepts, confidence, and attitudes using 10-point Likert scales. Quantitative data were analyzed using the Wilcoxon matched-pairs signed-rank and Fisher exact tests. Semistructured interviews explored participants’ experiences and helped to explain their quantitative responses. Interview transcripts were analyzed using thematic analysis.

**Results:**

Findings from the literature review and targeted needs assessment identified gaps in practical quality improvement education related to project design, implementation, and ethical considerations, which informed workshop co-design. In total, 31 learners attended the workshop, and 77.4% (24/31) completed preworkshop and postworkshop surveys. There was a significant improvement in participants’ understanding of the Plan-Do-Study-Act cycle (preworkshop median score 2.0, IQR 1.0‐2.8 vs postworkshop median score 4.0, IQR 4.0‐5.0; *P*<.001). Confidence in engaging in quality improvement projects improved significantly (preworkshop median score 4.5, IQR 2.3‐7.0 vs postworkshop median score 7.5, IQR 6.3‐8.0; *P*=.004). Self-reported knowledge of additional methodologies, including Six Sigma, Lean, and root cause analysis, also improved significantly. Participants rated the workshop highly (median score 9.5 out of 10). Qualitative findings indicated that participants perceived improved capability in project planning, greater ethical awareness, and stronger motivation to apply learning in clinical practice. These findings reflect self-reported learning experiences rather than objectively verified skill development.

**Conclusions:**

Learner co-design was successfully integrated within Kern’s curriculum development framework to develop a practical quality improvement workshop informed by identified learner needs. Participation in the workshop was associated with improved self-reported understanding, confidence, and positive perceptions of relevance and usefulness. Future research should examine longer-term outcomes and evaluate adaptation across broader educational settings.

## Introduction

Quality improvement (QI) is pivotal in enhancing patient safety, outcomes, and overall health care efficacy [1]. QI is defined as a structured, collaborative, and inclusive approach for sustained transformation and continuous improvement in health care [2]. Medical audits and quality improvement projects (QIPs) are the most common approaches for operationalizing QI principles in clinical practice. Medical audits systematically evaluate health care delivery against established standardized guidelines within a clinical setting [3], whereas QIPs focus on identifying areas for improvement and implementing targeted interventions to enhance the quality of care [4].

Integrating QI principles into medical education curricula is increasingly recognized as essential [5]. The General Medical Council mandates that medical graduates understand and apply QI principles in clinical practice [6]. Furthermore, participation in QI activities forms part of the annual professional revalidation process [7], through which licensed physicians demonstrate that they remain fit to practice in the United Kingdom. Despite its recognized importance, significant gaps remain in the integration of QI into medical education, with limited literature on the impact of QI education on learners [8].

Involving end users in curriculum development improves learning outcomes, motivation, and satisfaction [9,10]. Although examples of co-designed curricula in medical education are increasing, there is a paucity of co-designed QI education. To address this paucity, this study had three objectives: (1) to describe how learner co-design was operationalized within Kern’s 6-step curriculum development framework to develop a QI workshop for medical students and resident physicians; (2) to evaluate preworkshop and postworkshop changes in learners’ self-reported understanding of and confidence in QI; and (3) to explore participants’ attitudes toward QI and their perceptions of the workshop’s relevance to future practice.

## Methods

### Overview

The study was conducted between April 2024 and July 2024 at a UK university that serves approximately 2000 medical students annually. A mixed methods pre-post educational intervention design was used to evaluate a co-designed QI educational workshop [11]. The workshop was developed and delivered by a group of medical students working under the mentorship of a consultant clinician with expertise in QI. These individuals constituted the organizing committee responsible for designing and implementing the educational intervention.

We adopted Kern’s 6-step approach to curriculum development, informed by principles from Kolb’s Experiential Learning Theory, to guide the co-design and development of the workshop [12,13]. Each step is described in detail later to show how learner co-design informed the development process. To measure the impact of the workshop, we used a pre-post educational evaluation design with surveys and interviews. Quantitative data were used to assess changes in participants’ self-reported understanding, confidence, and attitudes, whereas qualitative data were used to explore how participants experienced the workshop and why particular elements were perceived as useful or challenging. The 2 data strands were collected sequentially and integrated at the interpretation stage using a convergent mixed methods approach, whereby the quantitative findings showed what changed after the workshop, and the qualitative findings helped explain why those changes may have occurred.

Participants included medical students from the University of Birmingham Medical School and resident physicians from the University Hospitals Birmingham National Health Service Foundation Trust who voluntarily registered to attend the workshop. Recruitment used a convenience sampling approach [14]. The event was advertised through university mailing lists, social media platforms, and institutional group messaging channels. Snowball sampling also occurred [15], as potential participants shared information about the workshop within their peer networks to encourage attendance.

### Ethical Considerations

This project underwent institutional review at the time of submission and was approved to proceed without the requirement for full ethics committee review, as it was deemed not to raise substantial ethical concerns (ERN_2545-May2024). Informed consent was obtained electronically from participants before completion of the preworkshop and postworkshop surveys. Verbal consent was also confirmed at the start of each interview recording. To protect privacy and confidentiality, data were pseudoanonymized with participant ID codes and stored securely in password-protected cloud storage accessible only to the research team. No participant compensation was provided, and participation was entirely voluntary.

### Problem Identification and General Needs Assessment

A targeted literature search was conducted by an author (CP) to identify previously reported needs for QI education in medical training. The search strategy is summarized in Multimedia Appendix 1. The search identified 134 records, of which 8 (6) were deemed relevant following screening [8,16-22]. The literature shows that students often lack understanding of QI and that targeted educational interventions can improve learners’ knowledge of and attitudes toward QI participation [8,16,17,19,21].

The organizing committee considered several potential approaches to addressing these educational gaps. Rather than proposing formal curriculum reform, which may require substantial institutional resources, the group identified student-led workshops as a feasible and scalable strategy for introducing QI principles to learners [22]. However, the group also recognized potential limitations associated with peer-led education, including the possibility that participants may provide more favorable feedback when educational sessions are delivered by peers. To mitigate this risk, all workshop content was reviewed by a consultant clinician with expertise in QI and clinical audit prior to delivery to ensure scientific accuracy and educational quality.

### Targeted Needs Assessment

Individuals who registered for the workshop were invited to complete a preworkshop survey (Multimedia Appendix 2). Participants provided informed consent prior to completing the survey through an electronic participant information and consent form. The survey included 10-point Likert-scale items assessing participants’ baseline knowledge, confidence, and attitudes toward QI activities, as well as their prior experience with audits and QIPs, and their expectations for the workshop. Open-ended questions were included to allow participants to elaborate on their responses and provide additional insights into their experiences and perceived learning needs. Responses to open-ended questions were analyzed thematically to identify common themes informing the development of the workshop curriculum (Multimedia Appendix 3).

Participants who completed the preworkshop survey were invited to participate in a semistructured interview to explore their experiences and perceptions of QI in greater depth (Multimedia Appendix 4). The interview guide was specifically developed by the research team to ensure that key topics were consistently addressed across participants. Interviews were conducted either via Zoom (Zoom Communications Inc) or in person, depending on participants’ accessibility. Audio recordings were obtained with participant consent prior to the start of each interview. Among the 27 participants who completed the preworkshop survey, 12 (44.4%) agreed to participate in the interview (n=10, 83.3% medical students and n=2, 16.7% resident physicians). Interviews lasted approximately 20 minutes. All recordings were transcribed verbatim while ensuring accuracy. Transcripts were reviewed and analyzed independently by 2 study authors (ALJY and MI) using thematic analysis [23]. Data were stored securely in password-protected institutional cloud storage accessible only to members of the research team.

Two researchers collaboratively developed a coding framework after familiarizing themselves with the interview transcripts. Discrepancies in coding were discussed and resolved through consensus to ensure consistency. Qualitative analysis was conducted using NVivo (version 14; Lumivero LLC). Intercoder reliability was assessed using the Cohen kappa coefficient [24]. Codes were inductively grouped into broader themes, which were subsequently reviewed and refined.

### Goals and Objectives

On the basis of the findings from the literature review and targeted needs assessment, the program goal and workshop learning objectives were developed to ensure that the final curriculum directly addressed the integration of learner co-design into the workshop’s development. The overall program goal was to equip early-career health care learners with foundational knowledge, skills, and confidence to engage in QI and medical audit activities in clinical practice.

The workshop was designed to achieve the following learning objectives: (1) to improve participants’ understanding of medical audits and QIPs, (2) to increase participants’ confidence in engaging with QI activities, and (3) to strengthen participants’ awareness of practical, ethical, and implementation issues relevant to QI work.

These outcomes were evaluated using self-reported preworkshop and postworkshop survey responses and qualitative interviews, which together addressed the study’s second objective.

### Educational Strategies

The workshop curriculum was designed using Kolb’s Experiential Learning Theory [13], which conceptualizes learning as a cyclical process involving 4 stages: concrete experience, reflective observation, abstract conceptualization, and active experimentation. Educational activities were structured to approximate these stages within the constraints of a 1-day workshop. The workshop agenda can be found in Multimedia Appendix 5.

### Implementation

#### Concrete Experience: Engaging With Practical QI Scenarios

Participants first engaged in small-group activities designed to simulate the early stages of QI project development. These sessions were facilitated by medical students with prior experience in conducting QIPs. Activities included identifying clinical problems, brainstorming potential QIP ideas, developing SMART (specific, measurable, achievable, relevant, and time-bound) project objectives, designing interventions using Plan-Do-Study-Act (PDSA) cycles, and considering potential data collection strategies and ethical considerations.

These activities provided participants with opportunities to engage with QI concepts through practical problem-solving rather than passive learning.

#### Reflective Observation: Feedback and Peer Learning

Following the group activities, participants engaged in structured reflection sessions facilitated by workshop leaders. Participants reviewed their proposed project ideas, discussed potential implementation barriers, and reflected on their decision-making processes.

The workshop also included presentations of completed audits and QIPs delivered by senior medical students and resident physicians. These presentations were followed by “question-and-answer” and “expert feedback” sessions from faculty members.

These activities supported reflective observation by encouraging participants to critically examine both their own proposed ideas and real-world QI implementation examples.

#### Abstract Conceptualization: Building Theoretical Understanding

Expert-led lectures were delivered by clinicians with expertise in QI, clinical governance, and medical education. These sessions introduced the theoretical foundations of QI methodologies and contextualized the practical activities.

Lecture topics included definitions and distinctions between audits, QIPs, and research; examples of successful QI initiatives in clinical practice; regulatory and institutional structures supporting QI activities; and ethical and practical considerations when conducting QIPs.

These sessions supported the abstract conceptualization stage of Kolb’s learning cycle by linking participants’ practical experiences to a broader theoretical framework.

#### Active Experimentation: Applying Learning in Clinical Practice

The final stage of Kolb’s experiential learning cycle involves applying newly acquired knowledge in real-world settings. Although full implementation of QI initiatives extends beyond the workshop itself, participants were encouraged to initiate or participate in audits and QIPs within their clinical training environments following the workshop.

Participants were also invited to present their QI work at subsequent workshops, thereby supporting continued engagement with QI initiatives and extending the experiential learning process beyond the initial educational intervention.

### Evaluation and Feedback

Participants were invited to complete a postworkshop survey (Multimedia Appendix 6) using the same 10-point Likert-scale measures as the preworkshop survey to evaluate changes in self-reported understanding, confidence, and attitudes toward QI. Participants also rated individual workshop components using a 5-point scale and provided an overall workshop rating using a 10-point Likert scale. Participants who completed the survey were invited to participate in follow-up interviews to explore their experiences in greater depth (Multimedia Appendix 7). Of the 31 participants who completed the postworkshop survey, 15 (48.4%) agreed to be interviewed. The interview adopted a semistructured format similar to the preworkshop interviews and lasted approximately 15 minutes.

Quantitative survey data were analyzed using Prism (version 10.3.0; GraphPad Software LLC). Participants who completed both preworkshop and postworkshop surveys were included in the analysis. Descriptive statistics for nonparametric continuous data are presented as median (IQR), while categorical data are summarized as n (%). Ordinal data derived from Likert scales were described with medians and IQRs, and the significance of the differences was assessed using the Wilcoxon matched-pairs signed-rank test. The Kruskal-Wallis test was used to identify the most efficacious aspects of the workshop, given the nonparametric nature of the data. The Fisher exact test was used to calculate *P* values for categorical data. Statistical significance was set at *P*<.05. Responses to the open-ended questions and postevent interviews were analyzed using the same approach as for the preworkshop survey (Multimedia Appendix 8).

## Results

### Participant Characteristics

In total, 31 delegates attended the event (n=29, 93.5% medical students and n=2, 6.5% resident physicians; n=24, 88.9% female). Attendees represented a range of ethnic backgrounds (Asian: n=17, 63%, White: n=6, 22.2%, and Black: n=4, 14.8%). Overall, 87.1% (27/31) participants completed the preworkshop survey (median age 21, IQR 21-22 years), and 77.4% (24/31) participants completed both preworkshop and postworkshop surveys.

### Preworkshop Needs Assessment Findings

#### Overview

Intercoder reliability was substantial, with a Cohen κ coefficient of 0.70. A summary of the coding framework and resulting themes is provided in Multimedia Appendix 9. Three primary thematic domains were identified.

#### Education and Skills Development in Audits and QIPs

Although most participants had some exposure to audits and QIPs during medical school, there was a consensus on the inadequacy of the practical training. The PDSA cycle was the most recognized QI tool. Participants expressed confidence in data collection but acknowledged limited knowledge of designing a QIP and had limited skills in data analysis:

We did it this year as part of a 4th year curriculum, and I feel like...we weren’t taught a lot about what it actually was by the medical school. Even though we did a project on it. I still think a lot of people don’t really understand why it’s important and why we need to, as HCPs [health care professionals], get involved in doing it.

[Participant 004]

#### Challenges and Solutions in Audits and QIPs

Key challenges included resource constraints, a lack of incentives, difficulty generating novel project ideas, and the time-consuming nature of the audit process. Participants proposed dedicated roles for QI, improved support systems, and the importance of acknowledging errors and seeking feedback. Table 1 shows the highlighted challenges and the corresponding examples.

**Table 1.** Challenges in conducting audits and quality improvement projects.

Challenges	Examples
Lack of incentive or motivation	“...most of the time quality improvement projects are carried out by doctors themselves taking the time out...hard to find an incentive to do that...” [Participants 003]
Lack of ideas	“...coming with like a unique idea of a QIP and trying to do something that hasn’t been done before.” [Participants 002]
Tedious process	“...when you first start, when you have your data collection tool...it doesn’t accurately represent what you want to get out of it...have to use a small proportion of the patients...go back and amend...” [Participants 010]
Mistake acknowledgment	“...to acknowledge that mistakes have been made...take over the ego aspect...” [Participants 005]
Funding	“...if you have to bring something new into the world or into the hospital, then it becomes challenging.” [Participants 001]

#### Patient Safety, Confidentiality, and Ethical Considerations

Participants highlighted the importance of acting in patients’ best interests, anonymizing data, and adhering to strict confidentiality protocols. Ethical considerations included avoiding bias, maintaining neutrality, and respecting patient autonomy. However, there was limited experience or expertise on how to achieve these:

...So abiding by like the trust confidentiality...Not taking any confidential information with you outside the trust, anonymizing the data to the best that you can...and patient safety. I guess it depends on what you’re doing. But if you’re changing like procedures and stuff, making sure that it’s safe to do so, and like getting permission from supervisors and talking to people that have a lot more expertise about if there are any patient concerns and stuff with whatever you’re trying to propose.

[Participant 008]

### Quantitative Postworkshop Findings

#### Knowledge and Skills

**Figure 1.**
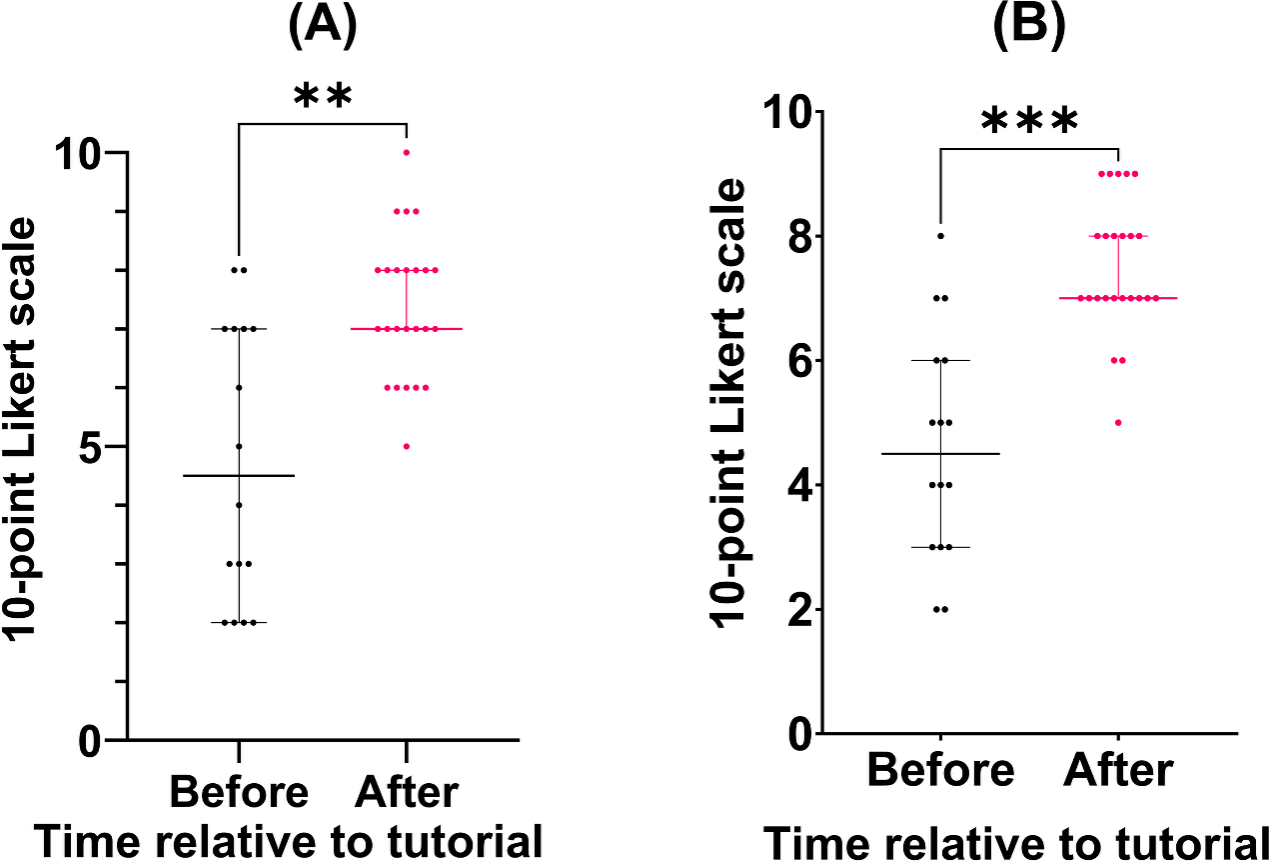
Changes in participants’ confidence scores for participating in a quality improvement project (QIP). (A) Confidence in participating in a QIP and (B) understanding of QIPs before and after the workshop. •⁠*P*<.05, **P<.01, ***P<.001. Horizontal lines are medians with 95% CIs.

Understanding of QIPs improved significantly (preworkshop median score 4.5, IQR 3.0-6.0 vs postworkshop median score 7.0, IQR 7.0-8.0; *P*<.001) as did confidence in participating in QIPs (preworkshop median score 4.5, IQR 2.3-7.0 vs postworkshop median score 7.5, IQR 6.3-8.0; *P*=.004; Figure 1). Self-reported understanding of various QI methodologies also improved significantly (Table 2).

**Table 2.** Change in understanding of specific quality improvement (QI) methodologies.

QI methodologies	Pre-event score, median (IQR)	Postevent score, median (IQR)	*P* value
Medical audits	3.0 (2.0-4.0)	4.0 (4.0-5.0)	<.001
Plan-Do-Study-Act cycle	2.0 (1.0-2.8)	4.0 (4.0-5.0)	<.001
Six sigma	2.0 (1.0-2.0)	3.0 (2.0-4.0)	<.001
Model of improvement	2.0 (1.0-2.0)	3.0 (2.3-4.0)	.001
Lean methodology	1.5 (1.0-2.0)	3.0 (2.0-3.0)	.002
Total quality management	1.5 (1.0-2.0)	3.0 (2.0-3.0)	.007
Root cause analysis	2.0 (2.0-3.0)	3.0 (2.0-3.8)	.03

#### Attitudes Toward QI

Participants felt that QI education is essential for improving patient outcomes and reported a high intention to apply the skills learned in the workshop. Barriers to implementing QIPs included limited knowledge, lack of support, time constraints, and insufficient resources.

#### Workshop Evaluation

Overall experience was rated highly (median score 9.5 out of 10). All components (lectures, hands-on activities, group discussions, and materials) were well received, with no significant differences in ratings. Participants emphasized the value of lectures, workshops, and oral presentations. Suggestions for improvement included broader participant inclusion, more frequent workshops, wider resource dissemination, and more expert speakers.

### Postworkshop Thematic Findings

#### Overview

Intercoder reliability was substantial, with a Cohen κ coefficient of 0.61. Four primary domains emerged from the postworkshop interviews. All codes and themes were summarized in Multimedia Appendix 10.

#### Positive Evaluation of the Workshop

Participants reported a substantially improved understanding of audits, QIPs, and implementation research. They valued the relevance of the workshop content, its delivery, and its alignment with their learning needs:

Overall, it was quite a positive experience, and I think the content felt very relevant. There was a lot of actionable stuff that was taught there, I think, and stuff I can use in the future to do audits and QIPs.

[Participant 002]

#### Knowledge and Skill Acquisition

Participants reported acquiring practical skills such as idea generation, objective setting, task allocation, and data analysis. They also gained a clearer understanding of the distinctions between audits and QIPs:

...made clear what is audit and what is QIP, and how it is different from the implementation research. I think now, I have a clearer idea of what is audit and what is QIP.

[Participant 001]

#### Recommendations for Future Participation and Access to QI Opportunities

Participants suggested improvements for future workshops, including broader participant inclusion, improved topic selection, and better signposting to QI opportunities and resources:

...like a dedicated sort of network, networking or signposting for opportunities out there, and how to get opportunities.

[Participant 003]

#### Ethical Awareness and Considerations

Participants highlighted the importance of patient confidentiality and informed consent in audits and QIPs. They also noted the complexity of ethics approval processes:

I was not aware of the ethical committees early on, but attending the conference helped me understand how projects are approved through the ethics committee. They also shared their experiences of the process, including that approval can take a couple of months. Overall, it gave me a better insight into these ethical issues.

[Participant 001]

## Discussion

### Principal Results

Co-design within a structured curriculum development model is feasible and may help produce a workshop that learners perceive as relevant, practical, and effective in building confidence. To our knowledge, this is among the few reported learner–co-designed QI workshops that integrate Kolb’s Experiential Learning Theory [13] into Kern’s curriculum development framework [12]. The workshop was associated with improved participants’ self-reported understanding and confidence, suggesting greater perceived readiness to engage in QIPs. Learner involvement in the design process may have helped ensure that the workshop addressed real-world learning needs, improved relevance, and promoted active engagement, which may be less evident in traditional didactic approaches.

Although experiential learning is fully realized through implementation of QI initiatives in real clinical environments, educational workshops can approximate key elements of this cycle through small-group activities, structured project design exercises, and facilitated reflection. In this workshop, participants engaged in simulated QIP planning and peer discussions, allowing them to apply theoretical concepts and reflect on potential implementation challenges before undertaking real-world QI initiatives.

Our findings are consistent with the broader literature synthesized by Peiris-John et al [8], which suggests that structured QI teaching can improve learner-level outcomes such as self-reported knowledge, confidence, and engagement. The workshop was similar to the interventions identified in that review because it combined multiple educational strategies, including didactic teaching, experiential learning, and small-group discussion. However, this study differed in that it evaluated a 1-day workshop that was co-designed with learners within Kern’s curriculum development framework and complemented by a qualitative evaluation of participants’ experiences [12].

Kern’s model was chosen for its practical [12] and stepwise approach to curriculum development, which aligns well with medical education, particularly when an intervention must progress from needs assessment to implementation and evaluation within a clearly structured and reportable framework. Its flexibility also supports refinement of educational programs in response to identified learner needs and feedback, and it has been widely applied in medical education [25]. Alternative approaches were considered but were less suitable for the aims of this study. For example, backward design is useful for aligning learning outcomes with assessment [26], but it places less emphasis on needs assessment and stakeholder-informed problem definition, both of which were central to the co-design process. Design-based research could also have been used because it supports iterative refinement in authentic settings [27]; however, it typically requires multiple cycles of testing and redesign and was therefore less feasible for a single workshop delivered within a limited time frame. Similarly, intervention development approaches primarily grounded in implementation science may have been useful for later scale-up [28], but they were less well suited to reporting the educational curriculum development process itself.

A convergent mixed methods design was chosen because it enabled triangulation of quantitative and qualitative findings, providing complementary insights into the educational intervention [29]. In this study, the quantitative component identified what changed after the workshop, particularly in participants’ self-reported understanding, confidence, and attitudes, whereas the qualitative component helped explain why these changes may have occurred by exploring participants’ experiences of the workshop. Alternative mixed methods approaches, including an explanatory sequential design, would typically require quantitative data collection and analysis to be completed before qualitative follow-up to explain specific findings, which was less practical within the time frame of a single workshop evaluation. An exploratory sequential design was also less appropriate, as this is generally used when qualitative findings are intended to inform the subsequent development of a quantitative instrument or intervention, which was not the purpose of this study.

This structured curriculum development process, based on Kern’s framework [12], supports transparency and reproducibility, which is a strength of the study. The workshop was co-designed with learners, which helped ensure that the educational content addressed identified learner needs and was perceived as relevant to practice. The mixed methods design strengthens the evaluation by combining quantitative and qualitative data, allowing the study to show not only what changed after the workshop, but also why participants perceived the intervention as useful and applicable. Finally, the workshop provides a practical, resource-efficient educational model that can be adapted to similar training contexts.

### Limitations

However, although the institution serves a large medical student population, this study evaluated a single voluntarily attended workshop using convenience and snowball sampling. As such, the sample was not intended to be statistically representative of the wider student population. These sampling approaches also limit the transferability of the findings to other settings and learner groups. A further limitation is that the evaluation primarily assessed self-reported understanding, confidence, and attitudes rather than objectively measuring knowledge acquisition or practical QI skills. The workshop placed greater emphasis on some QI approaches, particularly PDSA cycles, which meant that other methodologies were explored less extensively. In addition, longer-term follow-up examining whether participants subsequently implemented QI projects was not conducted due to logistical constraints and the limited study time frame.

Future research should explore the scalability of this model across multiple medical schools, assess its long-term impact on learners’ QI engagement in practice, and incorporate perspectives from a wider range of stakeholders, including faculty and institutional leaders. Future studies should also include more objective educational outcomes, such as preintervention and postintervention knowledge tests, case-based assessments, observed structured scenarios, rubric-based evaluations, or workplace-based assessments. In addition, future work could examine whether longitudinal one-to-one mentorship models support sustained engagement and facilitate the development of tangible scholarly outputs such as conference posters or oral presentations.

### Conclusions

The learner co-designed a QI workshop that improved understanding of QI concepts and increased self-reported confidence in engaging in QI activities, with positive perceptions of the workshop’s relevance and usefulness. Future research is needed to examine longer-term outcomes and assess whether this co-designed model can be adapted effectively across broader educational settings.

## Supplementary material

10.2196/83657 Multimedia Appendix 1 Search strategy for problem identification as part of Kern’s 6-step approach to curriculum development.

10.2196/83657 Multimedia Appendix 2 Early-career Physicians & Investigators Collaboration 2024 preworkshop survey.

10.2196/83657 Multimedia Appendix 3 Themes and codes developed through thematic analysis of the preworkshop survey.

10.2196/83657 Multimedia Appendix 4 Guide for preworkshop interview.

10.2196/83657 Multimedia Appendix 5 Program of events.

10.2196/83657 Multimedia Appendix 6 Postworkshop survey.

10.2196/83657 Multimedia Appendix 7 Postworkshop interview guide.

10.2196/83657 Multimedia Appendix 8 Themes and codes developed through thematic analysis of the postworkshop survey.

10.2196/83657 Multimedia Appendix 9 Themes and domains developed through thematic analysis of the preworkshop interviews.

10.2196/83657 Multimedia Appendix 10 Themes and domains developed through thematic analysis of the postworkshop interviews.

## Abbreviations

PDSA: Plan-Do-Study-Act
QI: quality improvement
QIP: quality improvement project
SMART: specific, measurable, achievable, relevant, and time-bound
