# Supplementary material for: Co-Designing and Evaluating a 1-Day Quality Improvement Workshop for Medical Students and Resident Physicians: Tutorial on Applying Kern’s Curriculum Development Framework
Source: JMIR Med Educ. 2026 Jun 17;12:e83657. doi: 10.2196/83657 (PMC13274911; doi:10.2196/83657)
Supplement: Multimedia Appendix 1 [file mededu-v12-e83657-s001.docx]

**Supplementary 1: Search strategy for problem identification as part of the Kern’s 6-step approach**

| **Database** | **Search Strategy** | **Filters** | **All** |
| --- | --- | --- | --- |
| EMBASE | #1 "Medical Students"/ or "medical student*".ti,ab. | None | 116 |
|  | #2 "Quality Improvement"/ or "quality improvement".ti,ab. or "QI".ti,ab. |  |  |
|  | #3 "Education, Medical"/ or "medical education".ti,ab. or "education".ti,ab. or "curriculum".ti,ab. |  |  |
|  | #4 "Projects"/ or "project*".ti,ab. or "program*".ti,ab. or "Audits"/ or "audit*".ti,ab. |  |  |
|  | #5 "Teaching"/ or "teaching".ti,ab. or "instruction".ti,ab. or "pedagogy".ti,ab. or "Mentors"/ or "mentorship".ti,ab. or "mentoring".ti,ab. |  |  |
|  | #6 "Evaluation Studies"/ or "evaluation".ti,ab. or "outcomes".ti,ab. or "assessment".ti,ab. |  |  |
|  | #7 1 and 2 |  |  |
|  | #8 7 and 3 |  |  |
|  | #9 8 and 4 |  |  |
|  | #10 9 and 5 |  |  |
|  | #11 10 and 6 |  |  |
| Medline | #1 "Medical Students"/ or "medical student*".ti,ab. | None | 58 |
|  | #2 "Quality Improvement"/ or "quality improvement".ti,ab. or "QI".ti,ab. |  |  |
|  | #3 "Education, Medical"/ or "medical education".ti,ab. or "education".ti,ab. or "curriculum".ti,ab. |  |  |
|  | #4 "Projects"/ or "project*".ti,ab. or "program*".ti,ab. or "Audits"/ or "audit*".ti,ab. |  |  |
|  | #5 "Teaching"/ or "teaching".ti,ab. or "instruction".ti,ab. or "pedagogy".ti,ab. or "Mentors"/ or "mentorship".ti,ab. or "mentoring".ti,ab. |  |  |
|  | #6 "Evaluation Studies"/ or "evaluation".ti,ab. or "outcomes".ti,ab. or "assessment".ti,ab. |  |  |
|  | #7 1 and 2 |  |  |
|  | #8 7 and 3 |  |  |
|  | #9 8 and 4 |  |  |
|  | #10 9 and 5 |  |  |
|  | #11 10 and 6 |  |  |
