# Supplementary material for: Co-Designing and Evaluating a 1-Day Quality Improvement Workshop for Medical Students and Resident Physicians: Tutorial on Applying Kern’s Curriculum Development Framework
Source: JMIR Med Educ. 2026 Jun 17;12:e83657. doi: 10.2196/83657 (PMC13274911; doi:10.2196/83657)
Supplement: Multimedia Appendix 2 [file mededu-v12-e83657-s002.docx]

**Supplementary 2: EPIC 2024 Pre-Workshop Survey**

Please click here to view the participant information and GDPR details. *

<https://docs.google.com/document/d/1F9jE4DcG_b7SjAVRnNQpAr-dqVWYKML3rFO-yEpR_G0/edit?usp=sharing>


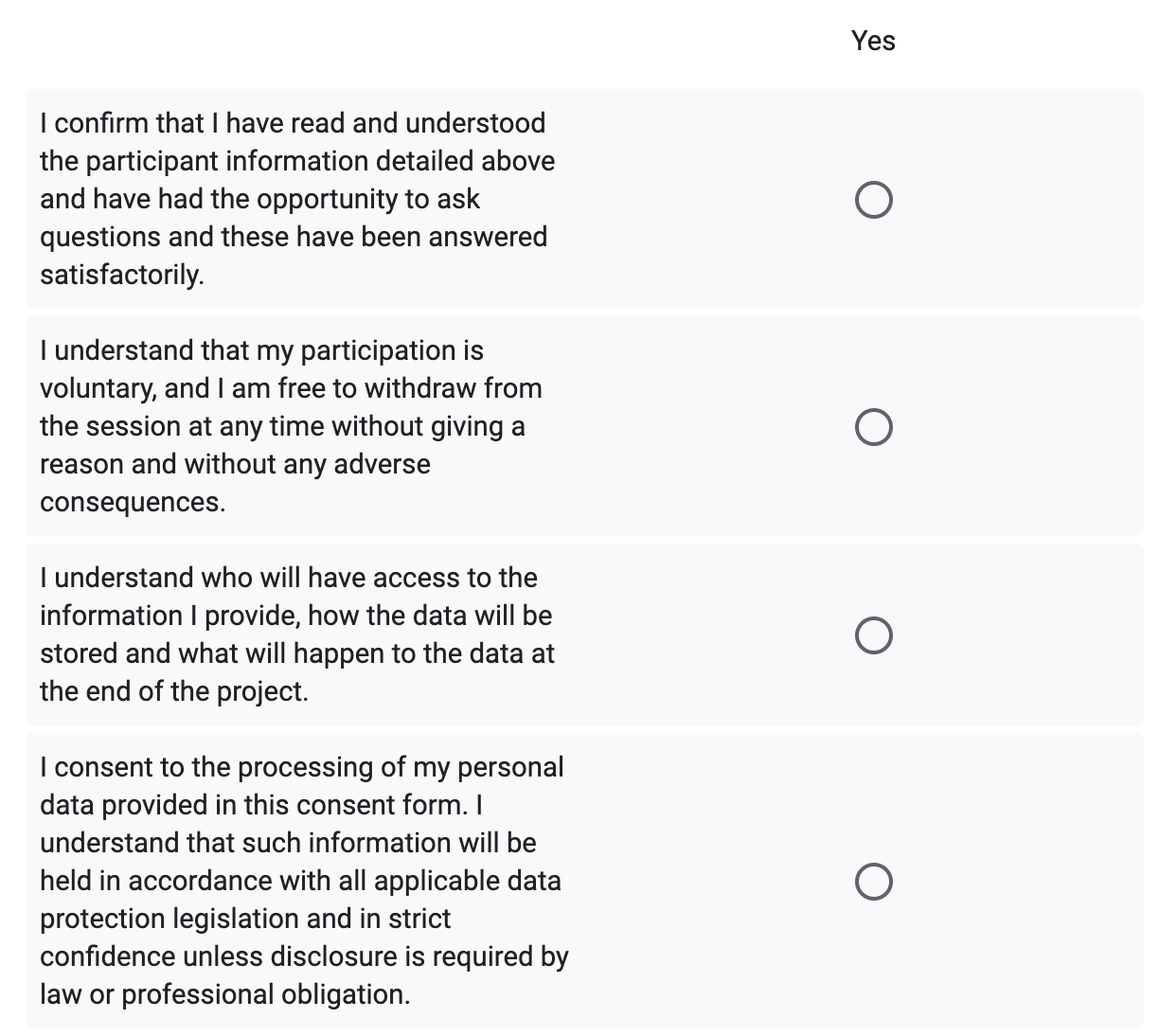


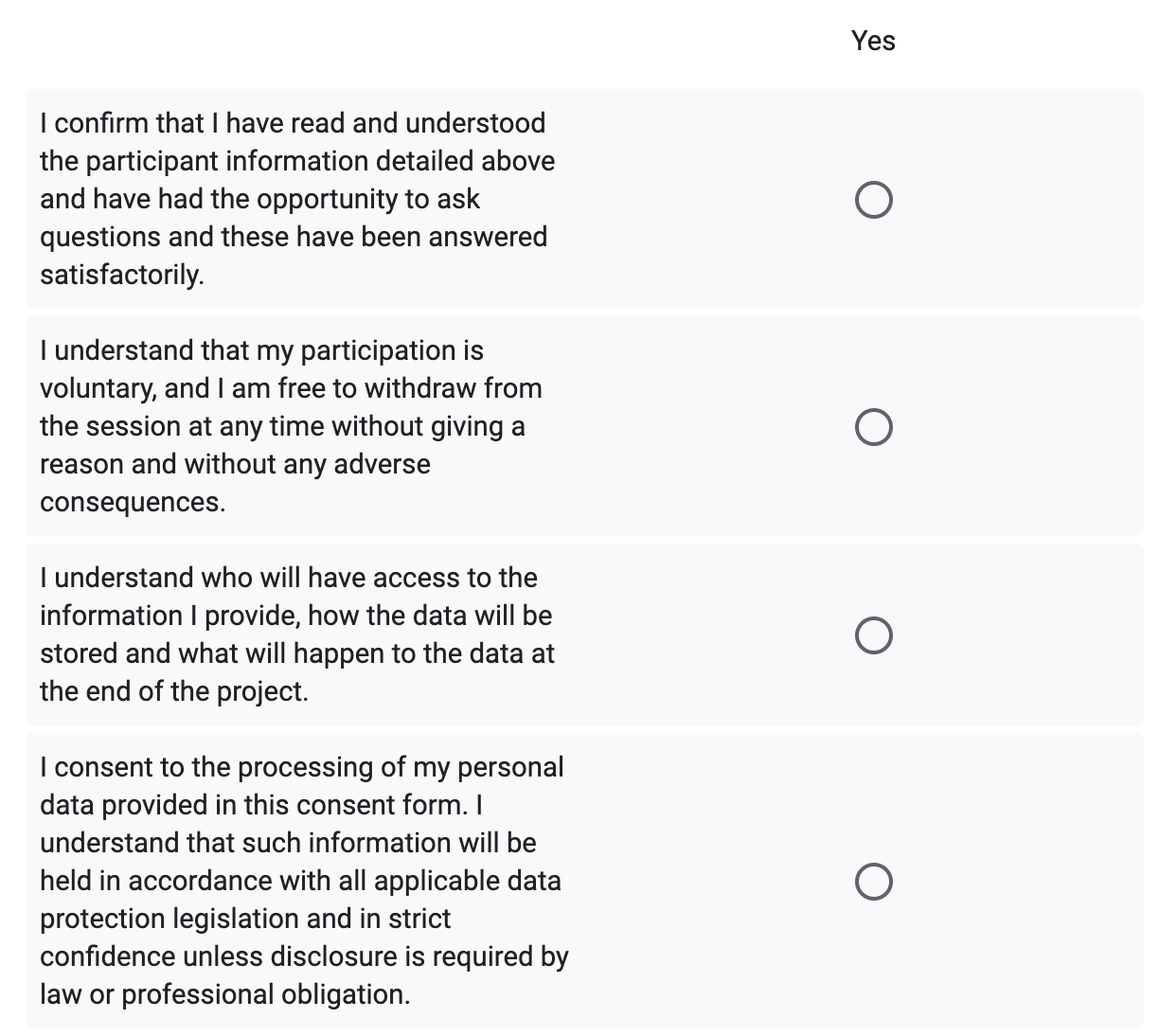

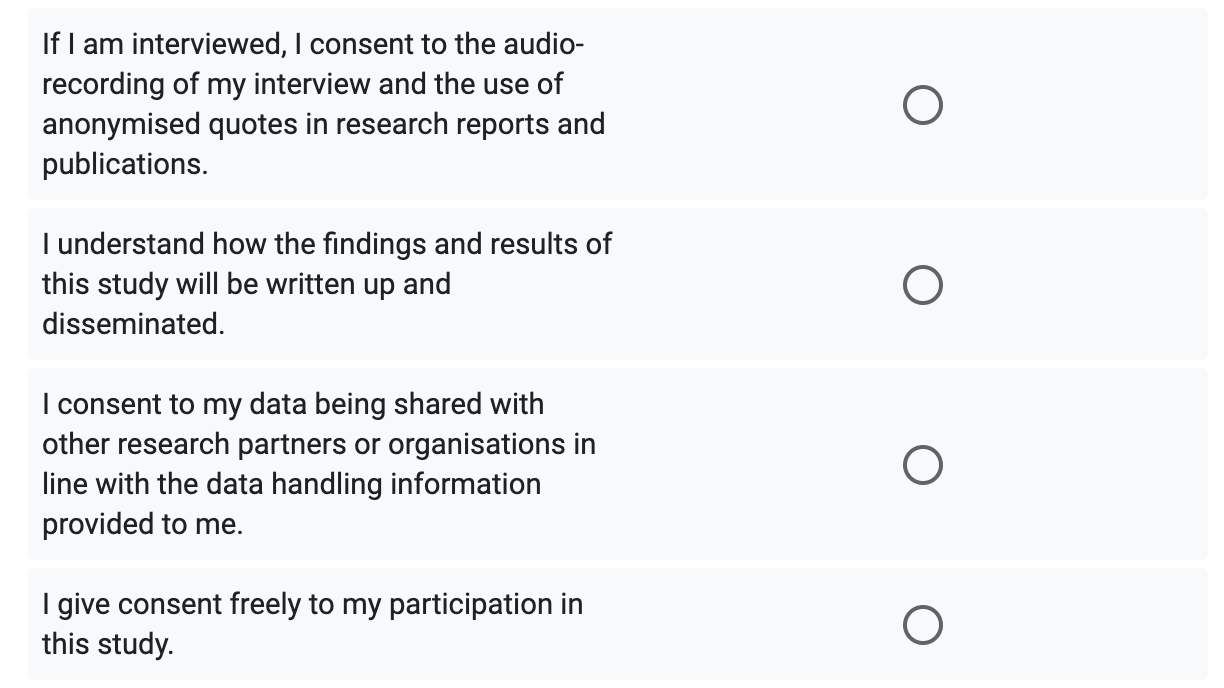


About You

First Name*:

Last Name*:

What is your current age in years? *:

What is your gender? *:


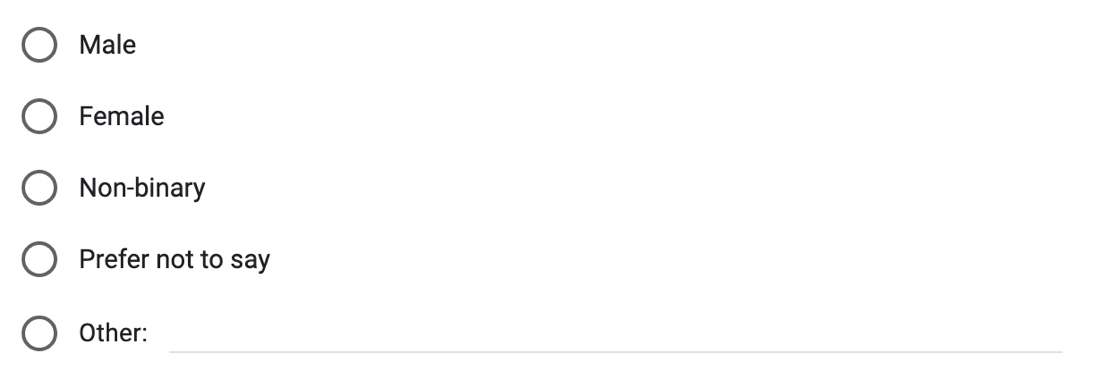


How would you describe your ethnicity? *:

Asian, Asian British or Asian Welsh

Black, Black British, Black Welsh of African background

Black, Black British, Black Welsh or Caribbean background

Mixed or Multiple ethnic groups

White

Other ethnic group

Prefer not to say

What is your current level of medical training? *:


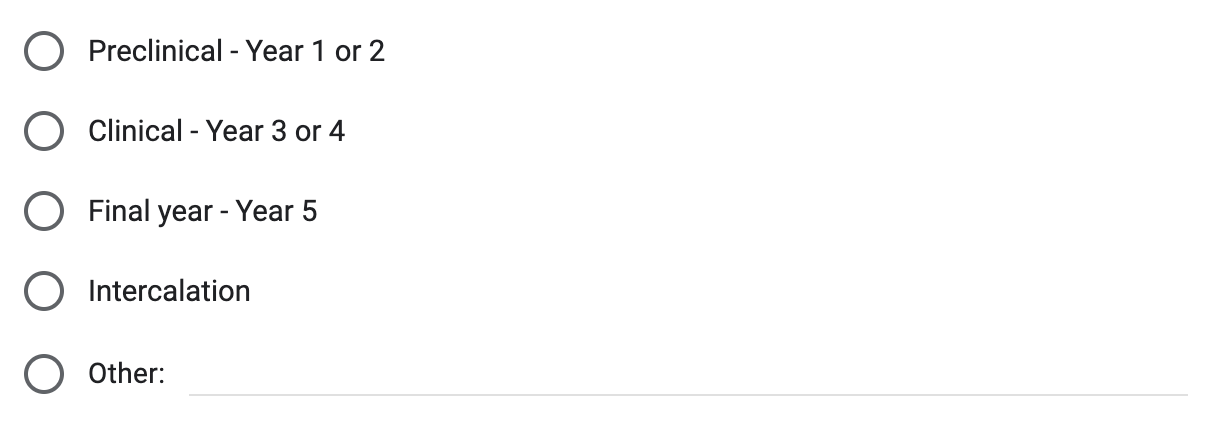


Have you previously participated in teaching session(s) or similar workshop(s) that is related to audits and QIPs? *


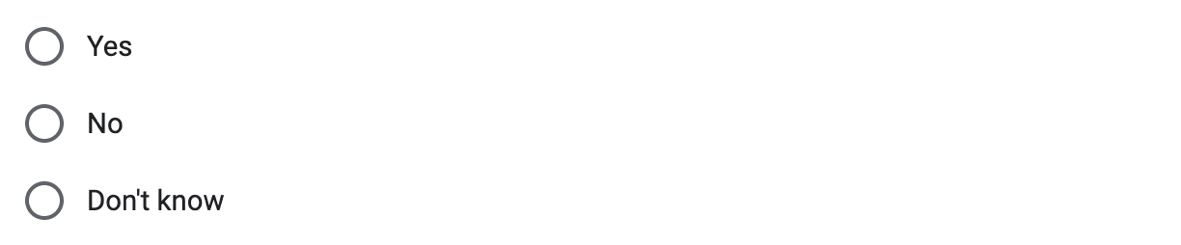


Knowledge and Experience

On a scale of 1 to 10, how would you rate your current understanding of Quality Improvement Projects (QIPs)? *


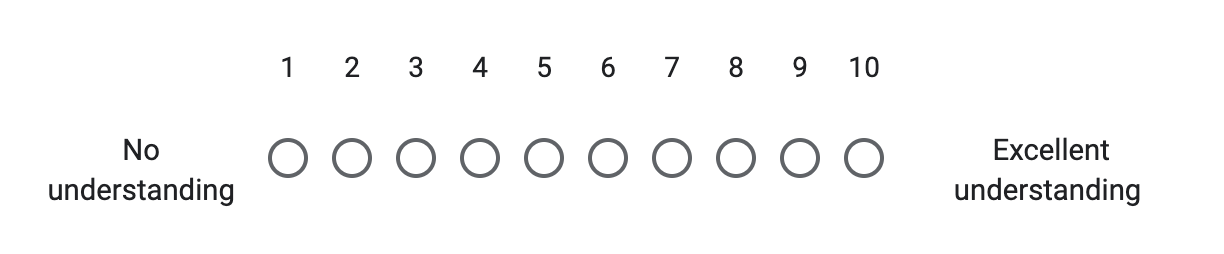


I have a good understanding of the following concepts: *


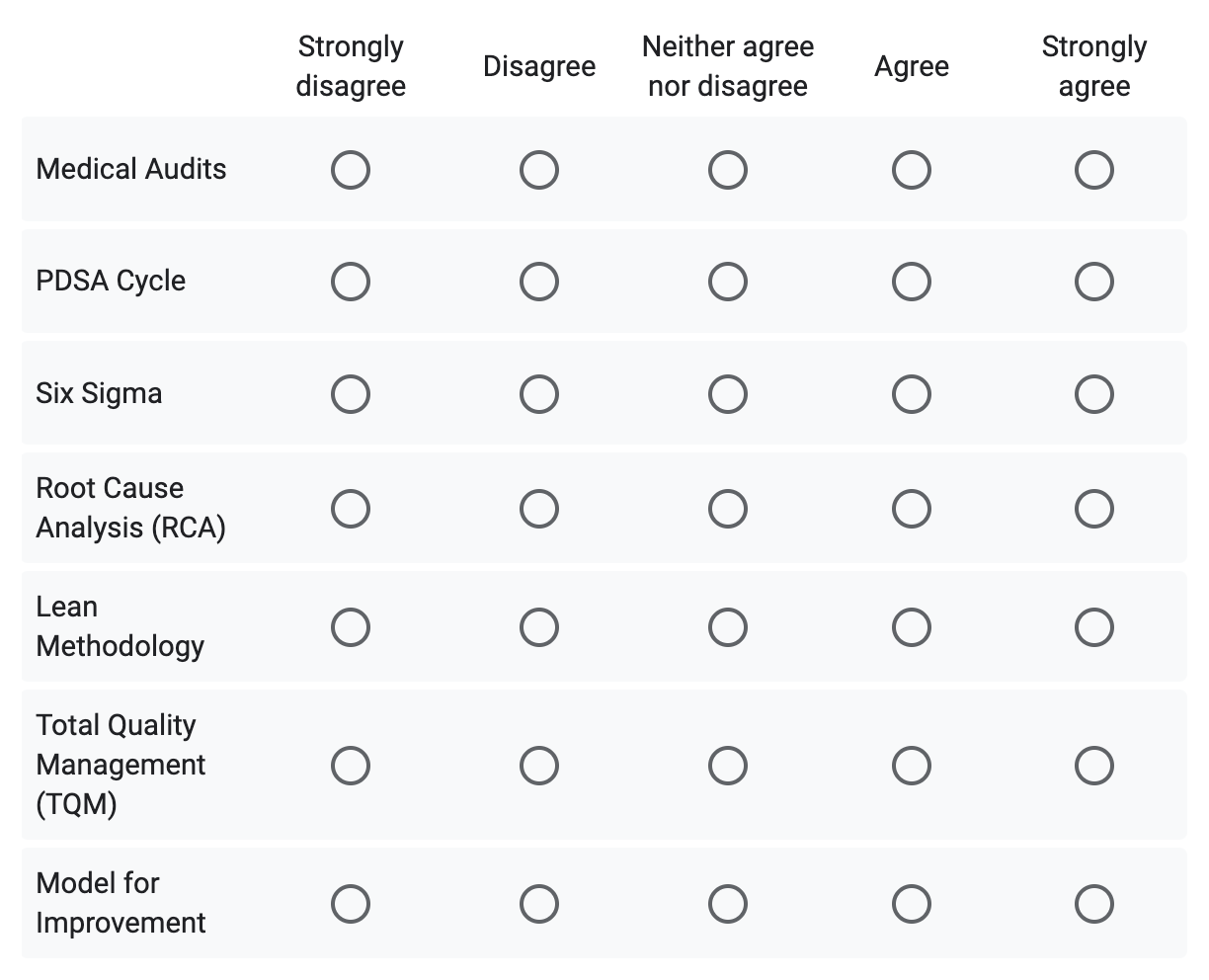


Skills and Confidence

On a scale of 1 to 10, how confident do you feel in your ability to participate in a quality improvement project or audit? *


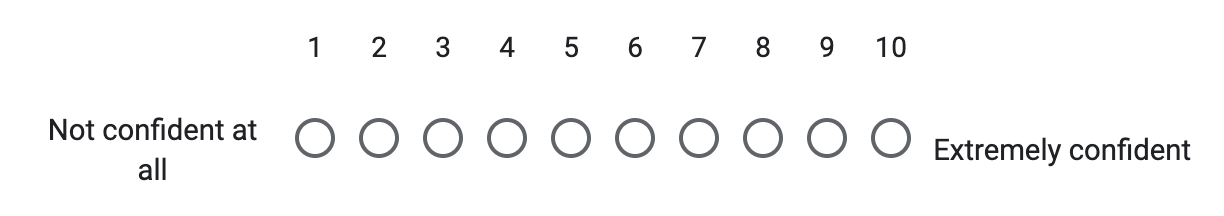


Please explain what factors influence your current level of confidence in participating in a quality improvement project or audit? *

Attitudes and Perceptions

To what extent do you agree with the following statements? (1 = Strongly disagree, 5 = Strongly agree) *


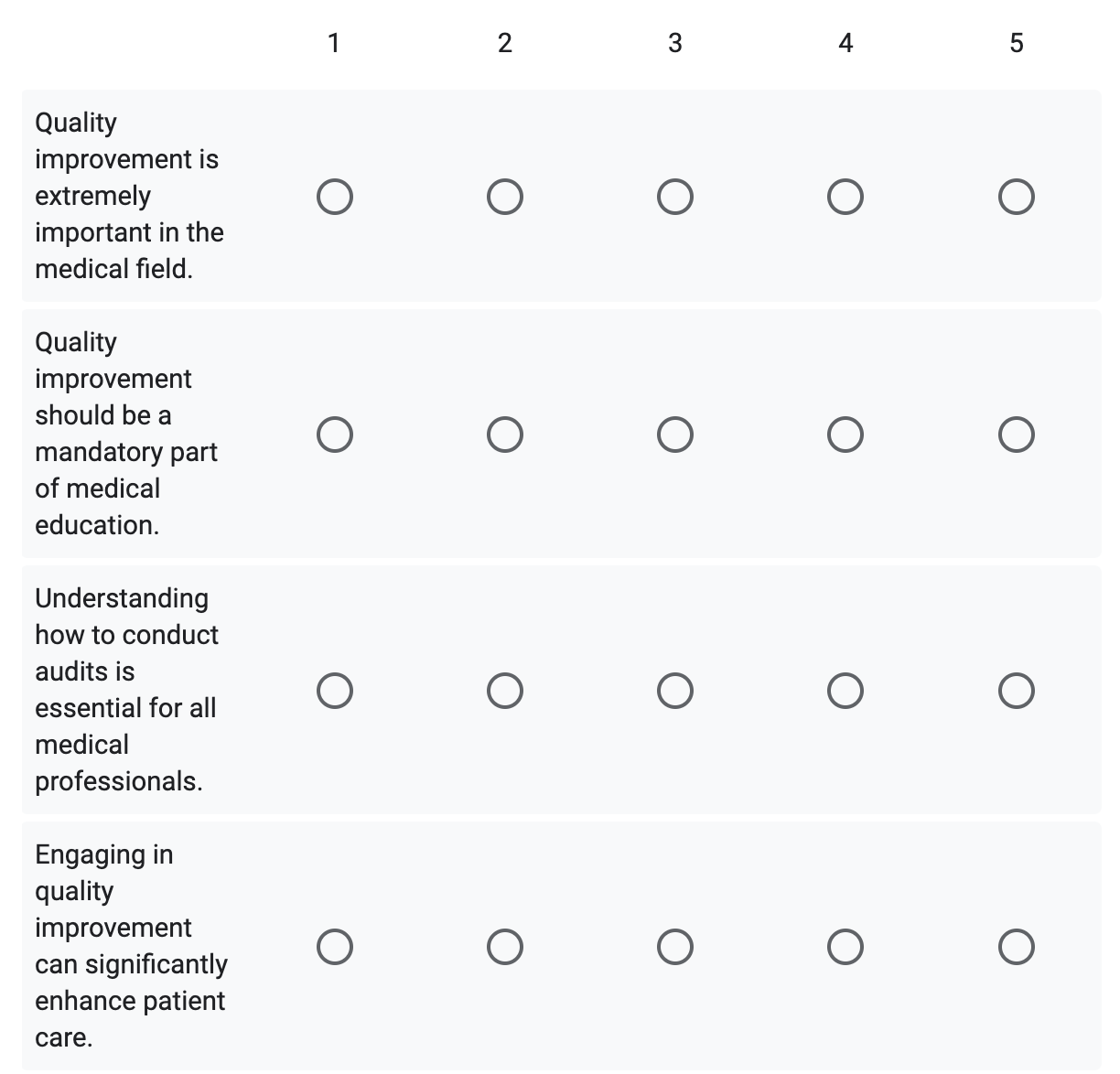


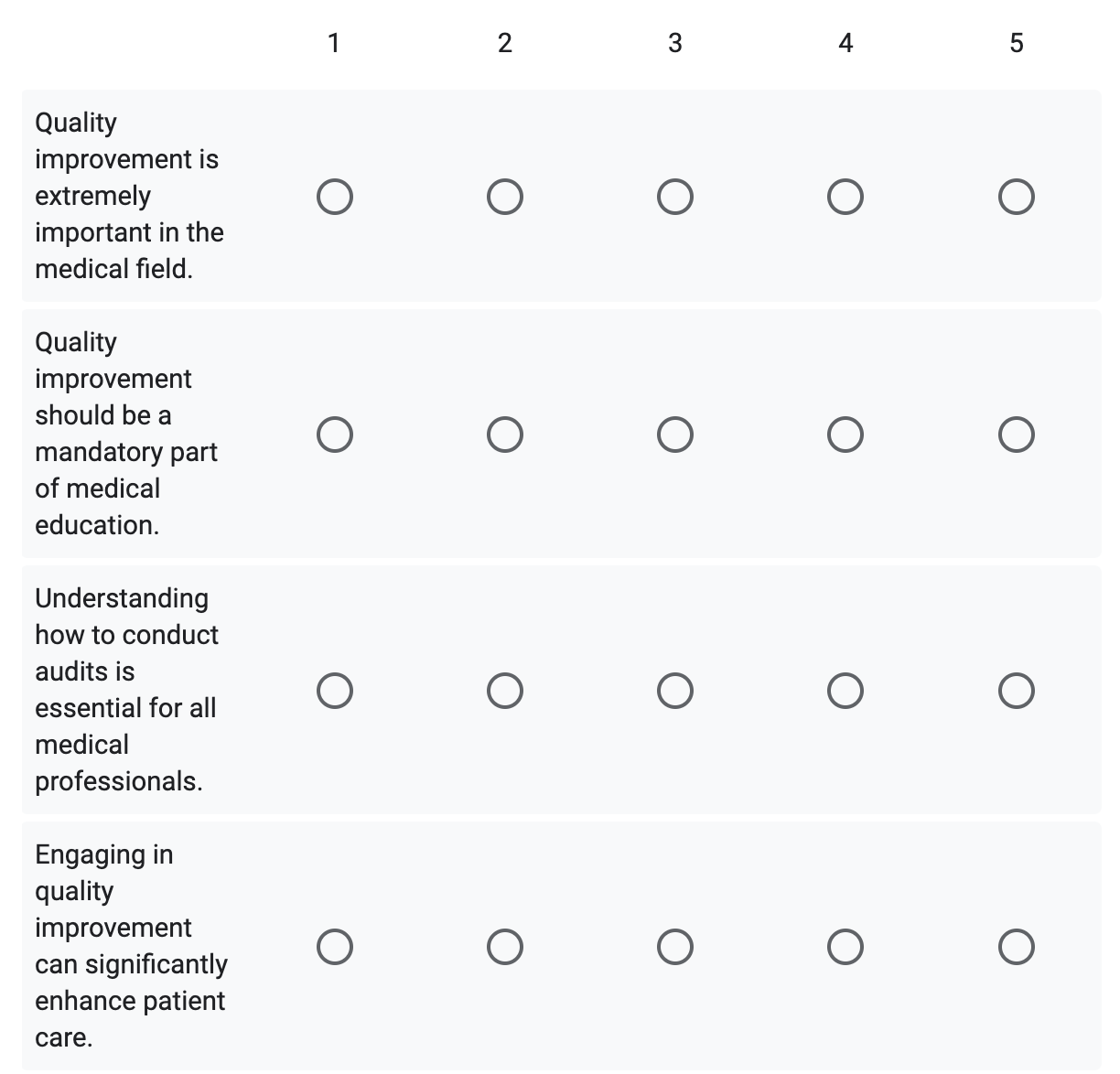


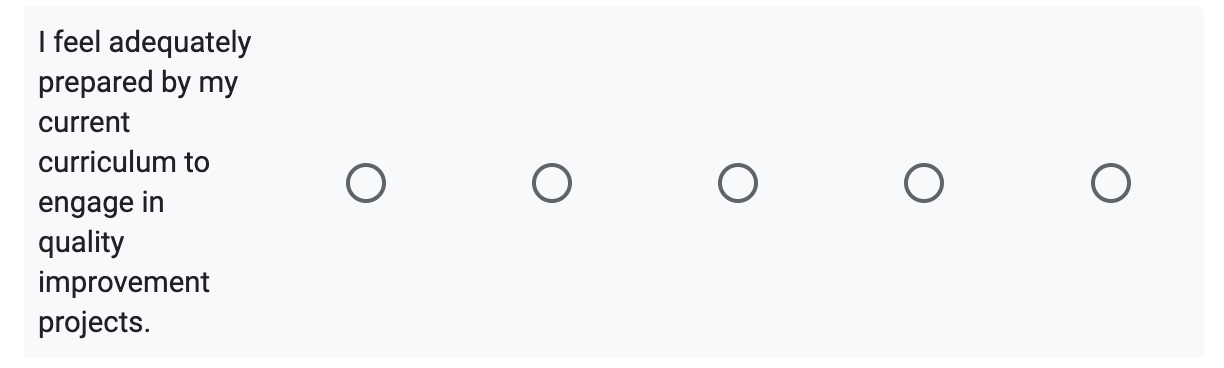


Application and Implementation

Do you feel adequately prepared to start or contribute to a quality improvement project or audit? *


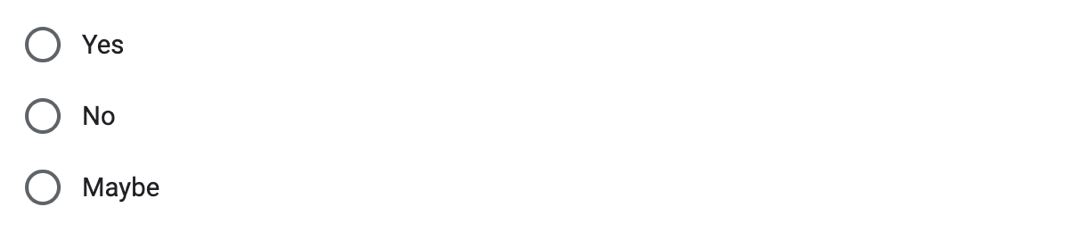


Please explain your answer to the previous question. *

On a scale of 1 to 10, how likely are you to apply the knowledge and skills gained from this workshop in your future medical practice? *


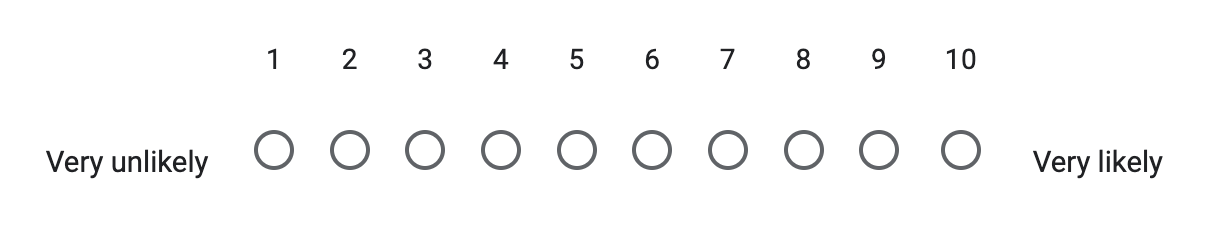


Expectations and Learning Objectives

What is your main objective(s) for attending this workshop? (Select all that apply) *


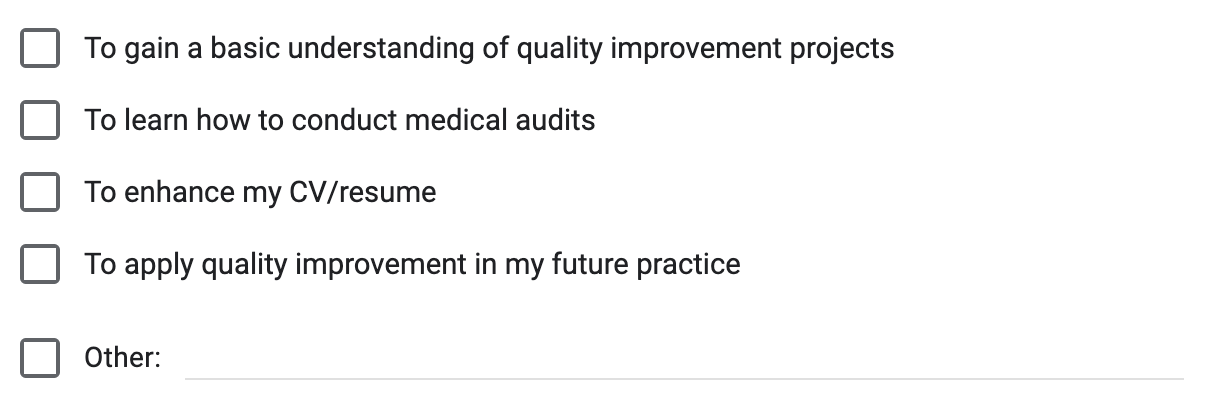


What do you perceive as the biggest barriers to implementing quality improvement projects in your current educational setting? (Select all that apply) *


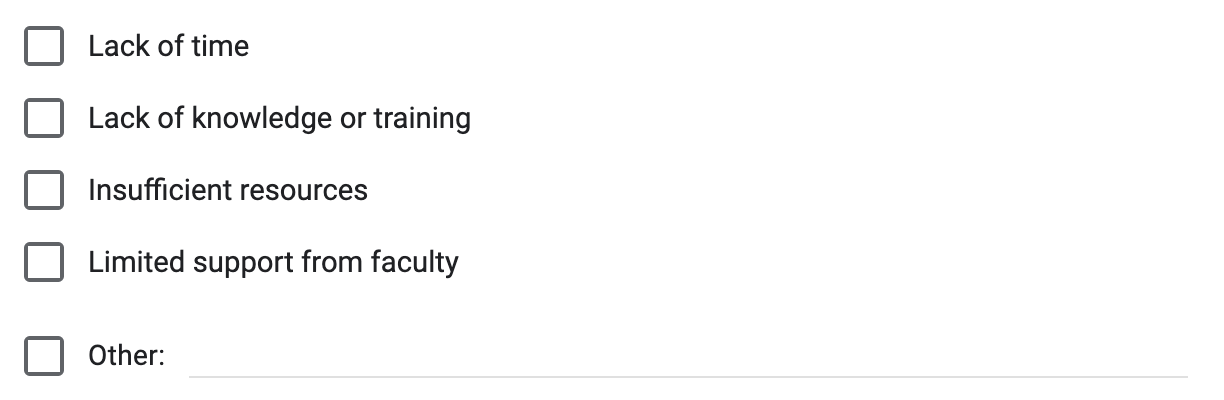


What do you think could be done to overcome these barriers? *

To what extent do you agree with the following statements? (1 = Strongly disagree, 5 = Strongly agree) *


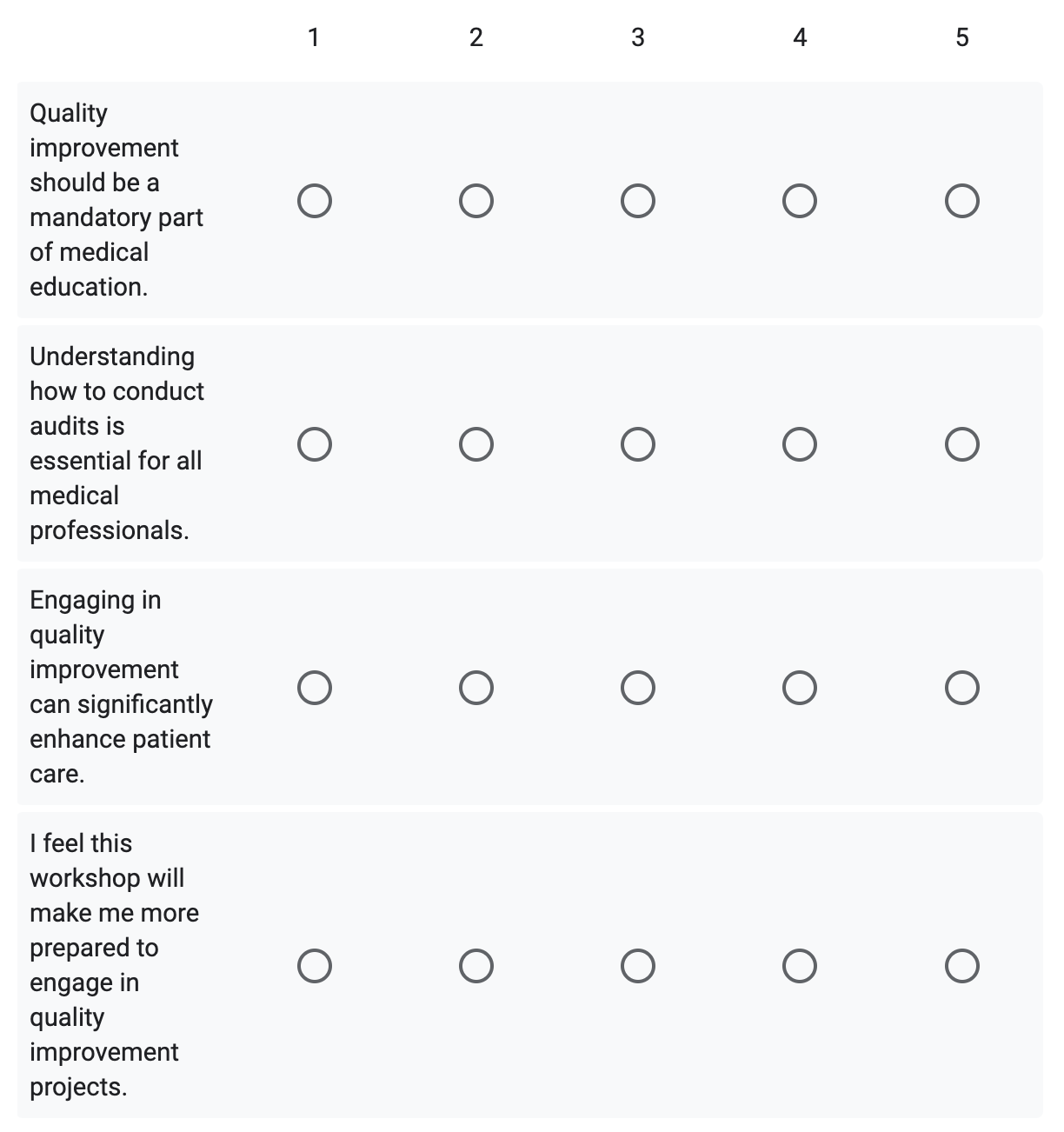


Do you have any specific topics or skills related to the audit or QIP that you would like to address during the workshop? (Select all that apply) *


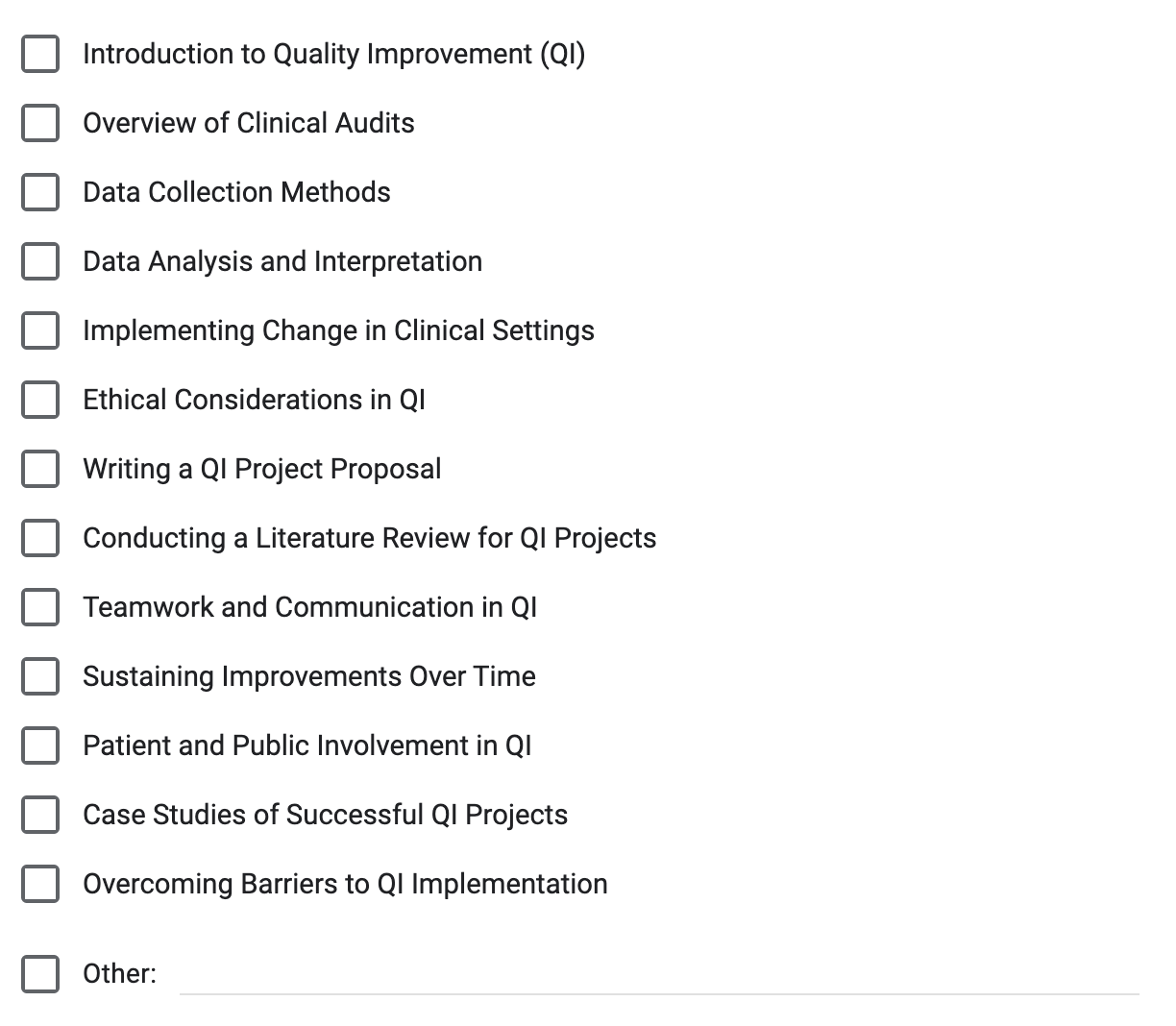


What support or resources would you need to better engage in quality improvement projects? *

Thank you for completing this survey. Rest assured any responses you have given us will be kept entirely confidential. Please click the finish button to submit your responses.
