# Supplementary material for: Co-Designing and Evaluating a 1-Day Quality Improvement Workshop for Medical Students and Resident Physicians: Tutorial on Applying Kern’s Curriculum Development Framework
Source: JMIR Med Educ. 2026 Jun 17;12:e83657. doi: 10.2196/83657 (PMC13274911; doi:10.2196/83657)
Supplement: Multimedia Appendix 3 [file mededu-v12-e83657-s003.docx]

**Supplementary 3: Themes and codes developed from thematic analysis from the pre-workshop survey**

| Excerpt | Code | Theme |
| --- | --- | --- |
| Lack of teaching | Lack of teaching | Factors influence your current level of confidence in participating in audits or QIPs before workshop |
| Lack of teaching about QIPs/audits and limited experience |  |  |
| Despite not having any experience, med school makes you an adaptive learner and I can pick up concepts quickly. That does mean I’d have to be guided initially and have the extra support, not everyone is able to provide that or is happy to do so. So confidence levels are more on the lower end of the spectrum. |  |  |
| Lack of teaching or explanation on these topics |  |  |
| I don't know where to start | Lack of experience |  |
| Not sure how to go about it |  |  |
| Lack of familiarity |  |  |
| Lack of experience |  |  |
| Less experience |  |  |
| Lack of experience |  |  |
| Lack of teaching about QIPs/audits and limited experience |  |  |
| I have only had experience of doing this once before, I feel like I’m only confident with the more simple forms of QIPs/audits |  |  |
| I don’t know where to turn to for opportunities. |  |  |
| Never had exposure |  |  |
| No prior teaching | Lack of knowledge | Feeling of adequacy in starting or contributing to a quality improvement project or audit before conference |
| I only know the basics of how audits work. I don’t know much about how to write a report up or data collection |  |  |
| I don’t know how to do it |  |  |
| I do feel prepared but it's good to have a formal training |  |  |
| I have had very limited teaching |  |  |
| I haven’t heard it prior to 3rd year and I don’t full understand what they are. | Lack of experience |  |
| Lack of familiarity |  |  |
| We have learned about the importance of audits through lectures but I still do not feel adequately prepared to take part in one myself, which is why I hope this workshop will help |  |  |
| still need more experience with QIPs |  |  |
| I know what it would involve in terms of choosing a topic and then collecting data, making a change and collecting data again etc but I’m not sure about what the exact logistics are in terms of liaising with a supervisor |  |  |
| I have no idea what’s expected of me or what I should be able to contribute. |  |  |
| More teaching | More teaching | Methods to overcome the barriers in implementing QIPs in current education setting |
| More sessions teaching how to produce audits |  |  |
| It should be part of internal medical teaching as well as registrar level educational teaching as many IMGs would not have formal training and experience. |  |  |
| More practical support by medical school |  |  |
| encouraging all students to do a both QIPs and audits uni hosting more conferences for students to practice saying them |  |  |
| Provide opportunities for students to conduct QIPs |  |  |
| building better networks to link students with doctors/QIPs/audits |  |  |
| Have the med school implement relevant workshops about this towards the end or the start of fourth year to place everyone on a similar level. |  |  |
| It would be nice to have a matchmaking event with investigators who have projects available to work on or even one with people interested in finding audit partners. |  |  |
| Come to the conference | Conference/workshops |  |
| More workshops such as this |  |  |
| Attend more workshops like this |  |  |
| More events like this and networking opportunities with faculty who are willing to take on students |  |  |
| Reach out more to other colleagues | Seek for senior support |  |
| Greater funding and time into these topics | Funding and time |  |
| More time |  |  |
| Written steps | Leaflets/handbooks | Support or resources suggested to better engage in QIPs |
| Leaflets or handbooks would be really useful |  |  |
| More information about how exactly students can get involved, who to contact if interested in doing a QIP |  |  |
| Workshops or booklets that provide a foundation of knowledge for students to feel more confident in approaching this topic |  |  |
| Booklet or leaflets |  |  |
| Booklets |  |  |
| Past student posters and experiences | Examples |  |
| Being able to access audits proposal from previous years |  |  |
| Support from the managers in hospital and accessibility to grants as funding poses a barrier | Support system |  |
| Practical support |  |  |
| more guidance on how to conduct them |  |  |
| More updates to free conferences and lectures | Conference/workshops |  |
| Workshop |  |  |
| Opportunities to become more widely available |  |  |
| Better learning resources on how to write a QI proposal, carry out a literature review, and collect and analyse data |  |  |
| Workshops or booklets that provide a foundation of knowledge for students to feel more confident in approaching this topic |  |  |
