# Supplementary material for: Co-Designing and Evaluating a 1-Day Quality Improvement Workshop for Medical Students and Resident Physicians: Tutorial on Applying Kern’s Curriculum Development Framework
Source: JMIR Med Educ. 2026 Jun 17;12:e83657. doi: 10.2196/83657 (PMC13274911; doi:10.2196/83657)
Supplement: Multimedia Appendix 4 [file mededu-v12-e83657-s004.docx]

**Supplementary 4: Guide for Pre-Workshop Interview**

Section 1: Introduction (5 mins)

1. Introduce the interviewer/facilitator
2. Thank participant for participating
3. Check participant’s audio and video are working
4. Confirm they are happy for us to record

START RECORDING

1. Introduce the interviewer/facilitator -Hi, I am Amanda, one of the facilitator for this research
2. Thank you for participating. Do you consent to the audio recording of your interview and using your anonymised quotes in research reports and publications?

Once they say yes, please continue with the below:

1. This interview is to understand medical students’ understanding of quality improvement projects and audits, and we would want to utilise this information to effectively address these specific needs and knowledge gaps so that future medical students can benefit from them.
2. There are no right or wrong answers. Your experiences and opinions are important and valid, and that is why we have invited you to speak with us today.
3. This interview will be kept confidential.
4. I have some questions I will ask to lead the conversation; otherwise, we want you to do the talking. If you think something is important, please bring it up, and if you don’t think a question or topic is relevant, please say so.
5. You can ask me to pause the recording at any time, and you are also free to leave the interview at any time.
6. Do you have any questions about the study itself or the interview?
7. If you want to find out more about how we handle your data, please refer to the participant information sheet that was emailed to you beforehand. If you have any questions about how we handle your data, please do ask me or get in contact with me via email.

Section 2: Background and Motivation

1. What inspired you to pursue a career in medicine?
2. Can you share an experience where you witnessed the impact of quality improvement in a healthcare setting?
3. What do you know about quality improvement projects in healthcare?
4. Why are you interested in attending this workshop on quality improvement and audits?

Section 3: Knowledge and Experience 

1. Have you participated in quality improvement projects or audits during your medical education? If so, can you describe your role and the outcomes?
2. What key principles of quality improvement in healthcare that you are familiar with?
3. Can you explain what an audit is and its importance in the healthcare field?
4. Have you ever used quality improvement tools or methodologies, such as Plan-Do-Study-Act (PDSA) cycles or Six Sigma? If so, please describe your experience.
5. What support or resources do you feel would be beneficial for medical students like yourself to enhance your understanding and skills in audits and QIPs?
6. Do you have any suggestions or feedback on how medical schools can better address students' needs and knowledge gaps in this area?

Section 4: Skills and Competencies

1. How comfortable are you with data collection and analysis as part of quality improvement initiatives?
2. What skills do you possess that you think will be beneficial for participating in quality improvement projects?
3. Can you discuss your experience with teamwork and collaboration in a clinical setting?

Section 5: Expectations and Goals

1. What do you hope to achieve by participating in this workshop?
2. Are there specific areas of quality improvement or audit processes you are particularly interested in learning more about?
3. How do you plan to apply the knowledge and skills gained from this workshop in your future medical practice?

Section 6: Challenges and Problem-Solving

1. Can you describe how you addressed a challenging situation you encountered in a clinical setting?
2. What are the biggest challenges to implementing quality improvement projects in healthcare, and how would you propose overcoming them?

Section 7: Reflection and Improvement

1. Can you reflect on a time when a healthcare process could have been improved and how you would have approached it?
2. How do you handle constructive criticism and use it for personal and professional growth?

Section 8: Ethical and Professional Considerations

1. How do you ensure that patient safety and patient confidentiality are maintained during quality improvement projects?
2. What ethical considerations do you think are important in the context of medical audits?

Finish Recording.
