# Supplementary material for: Co-Designing and Evaluating a 1-Day Quality Improvement Workshop for Medical Students and Resident Physicians: Tutorial on Applying Kern’s Curriculum Development Framework
Source: JMIR Med Educ. 2026 Jun 17;12:e83657. doi: 10.2196/83657 (PMC13274911; doi:10.2196/83657)
Supplement: Multimedia Appendix 5 [file mededu-v12-e83657-s005.pdf]

EPIC EARLY-CAREER PHYSICIANS & INVESTIGATORS CONFERENCE

Programme of events

	**Friday, 28th June 2024**
0900-0915	Registration
0915-0930	Welcome note/Introduction
0930-0945	Keynote speaker 1
0945-1000	
1000-1015	QIP Workshop 1
1015-1030	
1030-1045	
1045-1100	
1100-1115	
1115-1130	Refreshment Break
1130-1145	Oral Presentations
1145-1200	
1200-1215	
1215-1230	
1230-1245	
1245-1300	
1300-1315	Lunch
1315-1330	
1330-1345	
1245-1400	
1400-1415	Keynote Speaker 2
1415-1430	
1430-1445	QIP Workshop 2

Supported by

Society for Endocrinology

UNIVERSITY OF BIRMINGHAM

IMSR INSTITUTE OF METABOLISM AND SYSTEMS RESEARCH

EPIC EARLY-CAREER PHYSICIANS & INVESTIGATORS CONFERENCE

1445-1500	
1500-1515	
1515-1530	
1530-1545	
1545-1600	Refreshment Break
1600-1615	QIP Presentations
1615-1630	
1630-1645	Prizes and Closure

Supported by

Society for Endocrinology

UNIVERSITY OF BIRMINGHAM

IMSR
INSTITUTE OF METABOLISM AND SYSTEMS RESEARCH
