# Supplementary material for: Co-Designing and Evaluating a 1-Day Quality Improvement Workshop for Medical Students and Resident Physicians: Tutorial on Applying Kern’s Curriculum Development Framework
Source: JMIR Med Educ. 2026 Jun 17;12:e83657. doi: 10.2196/83657 (PMC13274911; doi:10.2196/83657)
Supplement: Multimedia Appendix 6 [file mededu-v12-e83657-s006.docx]

**Supplementary 7: Post-workshop survey**

EPIC 2024 Post-Workshop Survey

Please complete this questionnaire with spontaneous answers to reflect your true response. The information will be anonymised during analysis.

A certificate of participation will be sent to the email you have provided after completion of this evaluation.

Email*:

Certification

Name (as you would like it to be on the participation certificate) *:

Knowledge and Experience

On a scale of 1 to 10, how would you rate your current understanding of Quality Improvement Projects (QIPs)? *


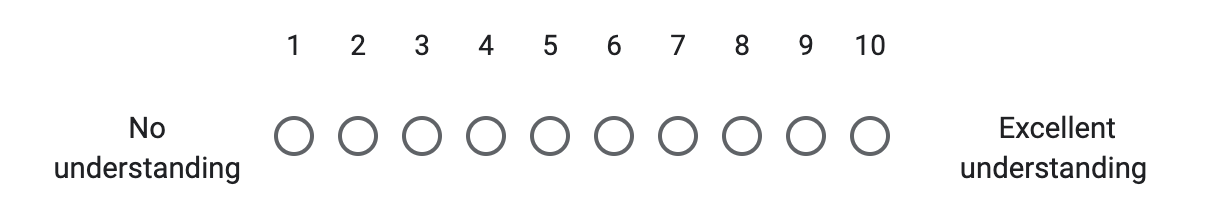


I have a good understanding of the following concepts*:


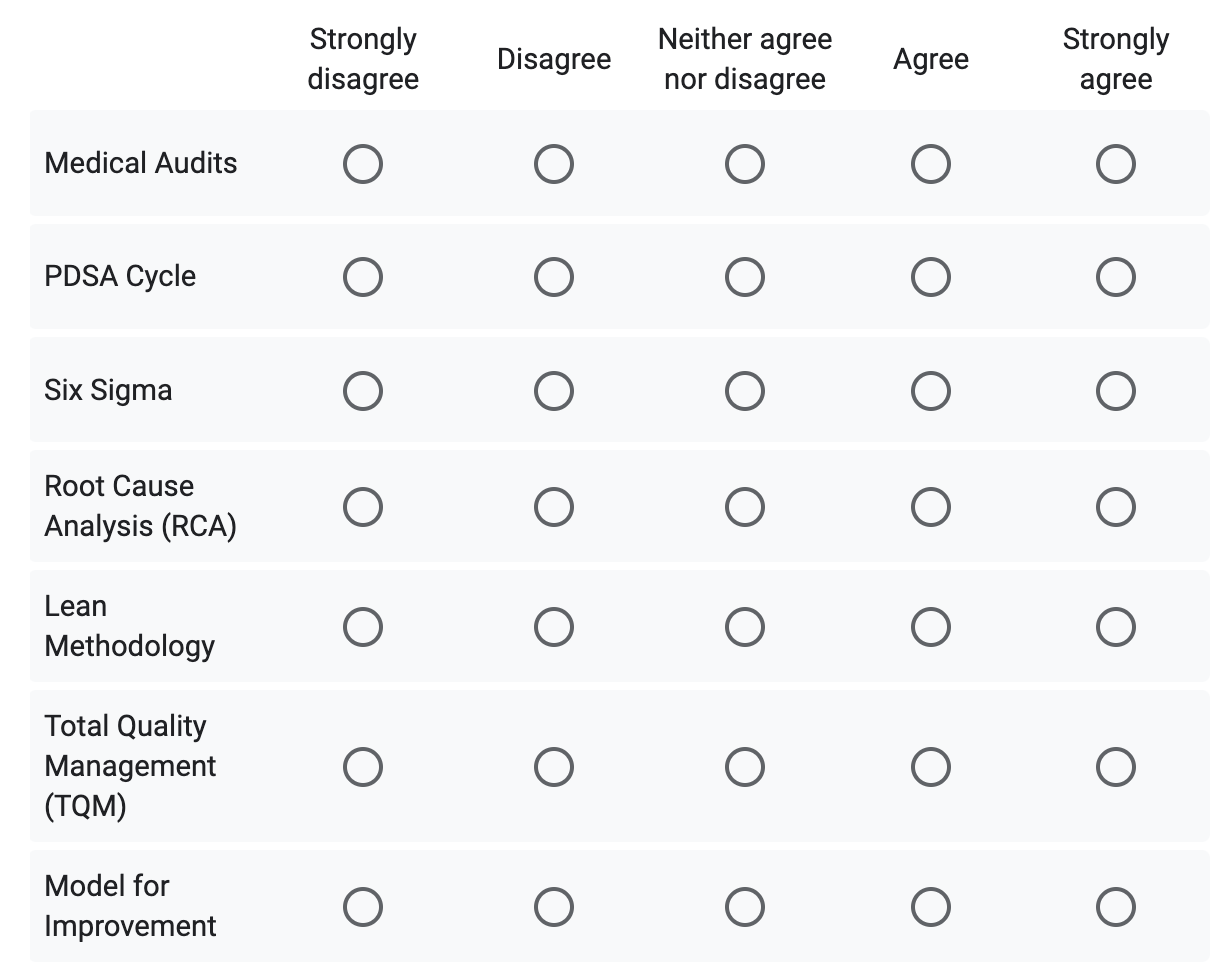


Skills and Confidence

On a scale of 1 to 10, how confident do you feel in your ability to participate in a quality improvement project or audit? *


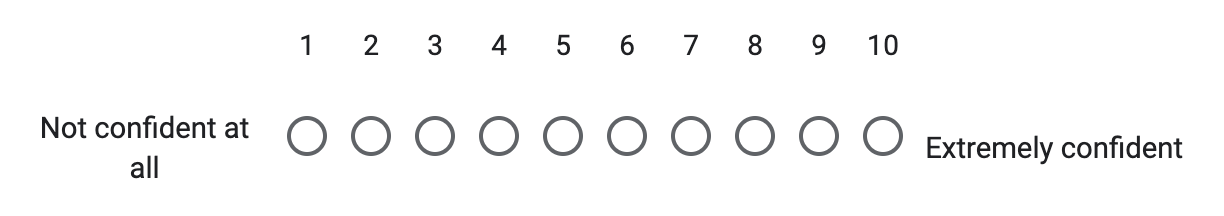


Please explain what factors influence your current level of confidence in participating in a quality improvement project or audit? *

Attitudes and Perceptions

To what extent do you agree with the following statements? (1 = Strongly disagree, 5 = Strongly agree) *


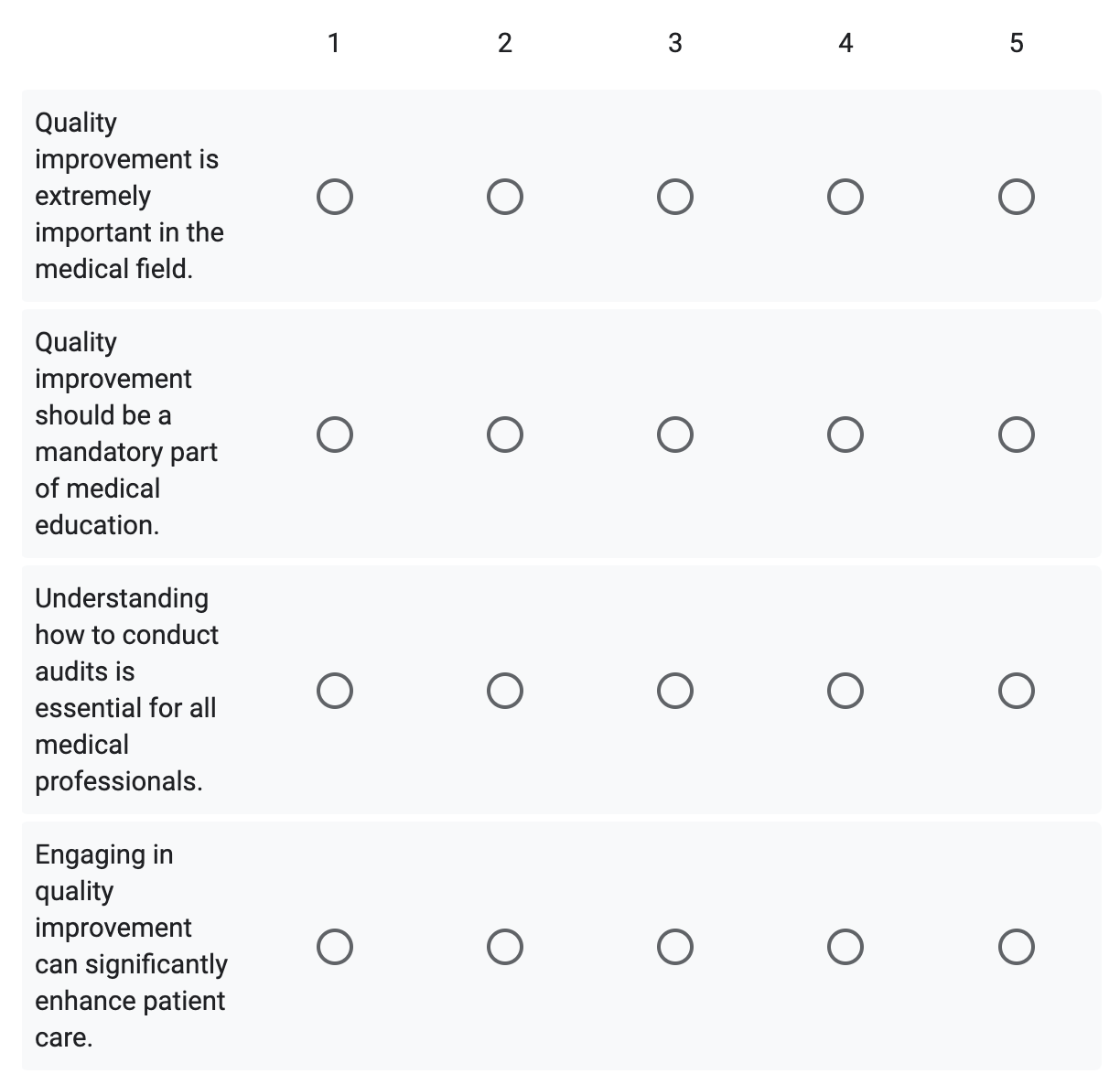


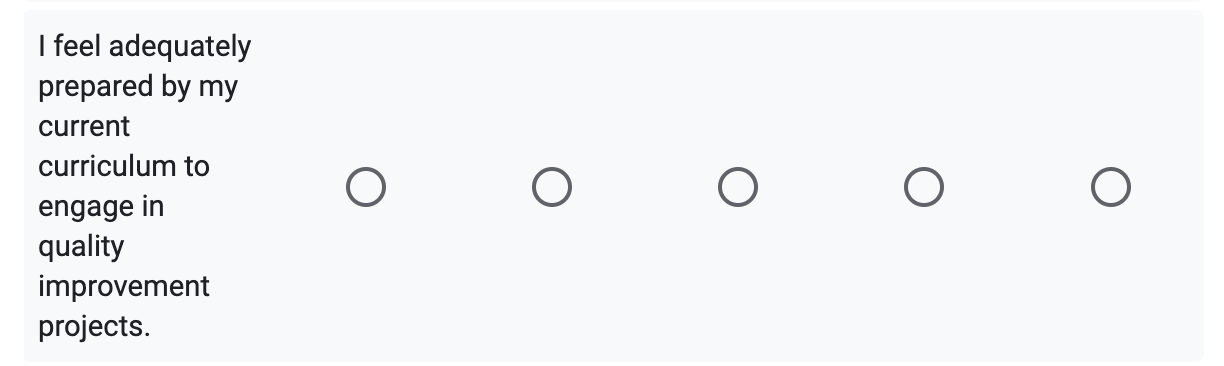


Application and Implementation

Do you feel adequately prepared to start or contribute to a quality improvement project or audit? *


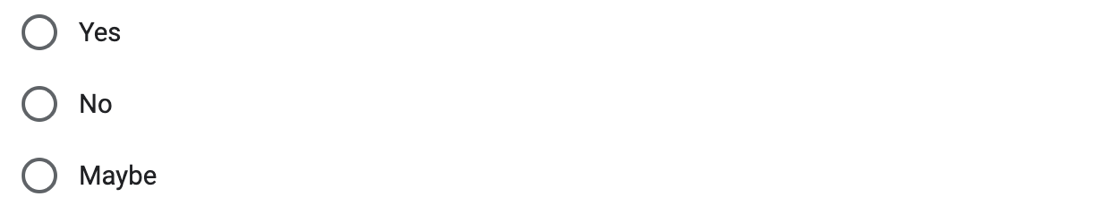


Please explain your answer to the previous question. *

On a scale of 1 to 10, how likely are you to apply the knowledge and skills gained from this workshop in your future medical practice? *


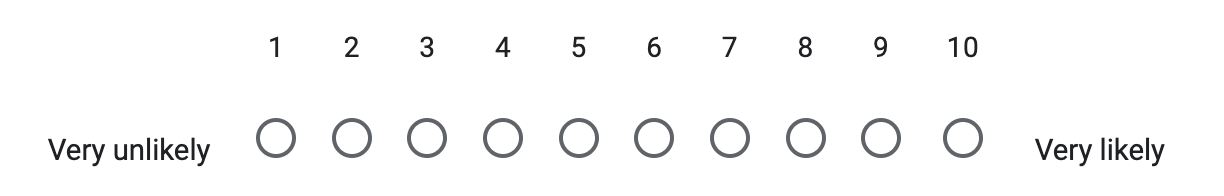


Workshop Content and Delivery

How effective were the following aspects of the workshop? (1 = Very ineffective, 5 = Very effective) *


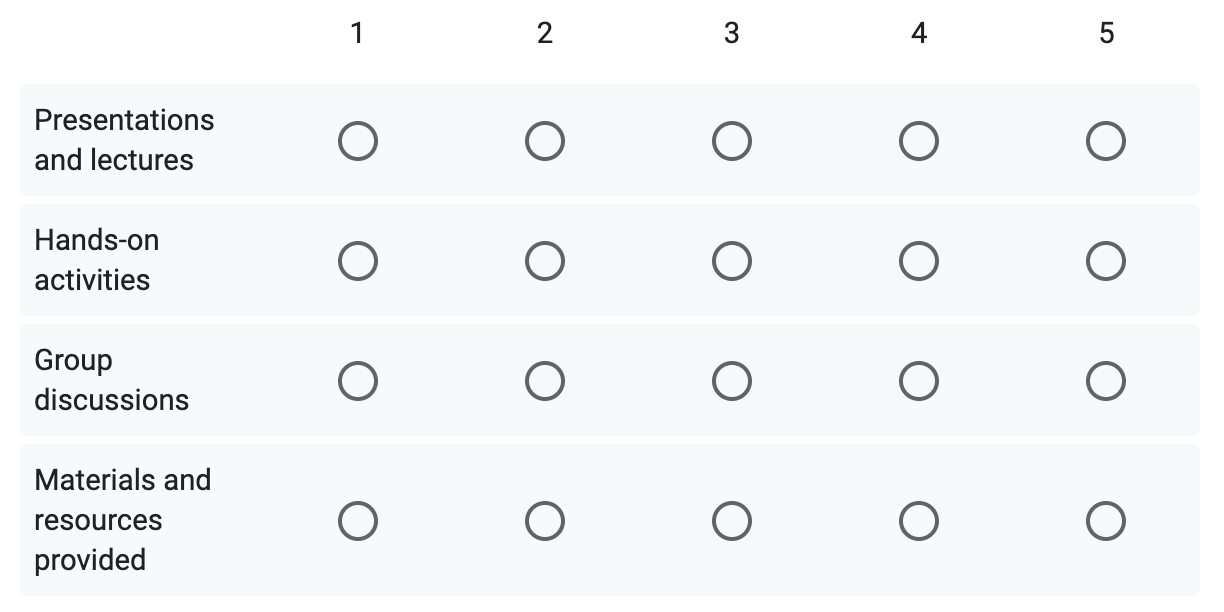


What aspects of the workshop did you find most valuable? *

What aspects of the workshop did you find least valuable? *

How do you plan to apply what you have learned in this workshop to your medical education or future practice? *

Do you have any suggestions for improving this workshop in the future? *

Any additional comments or feedback? *

Oral Communications

Did the format of the oral communications work for you in terms of *


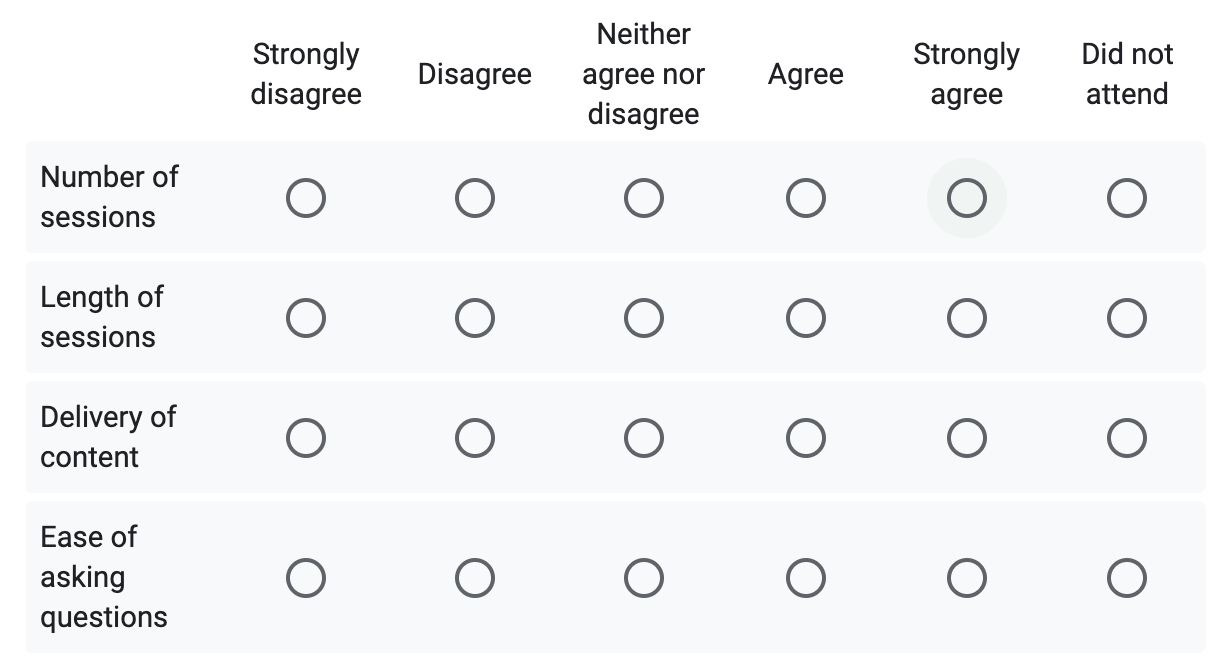


Invited Talk

Please rate the talk “Practical tips when it comes to doing efficient audits and QIPs” by x (1 = poor, 10 = excellent). *


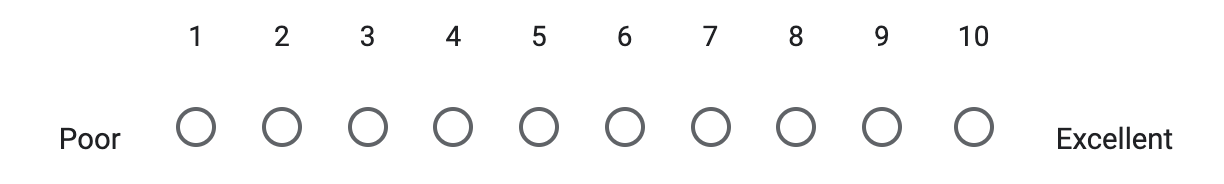


Conference

How would you rate the following? (1 = poor, 5 = excellent) *


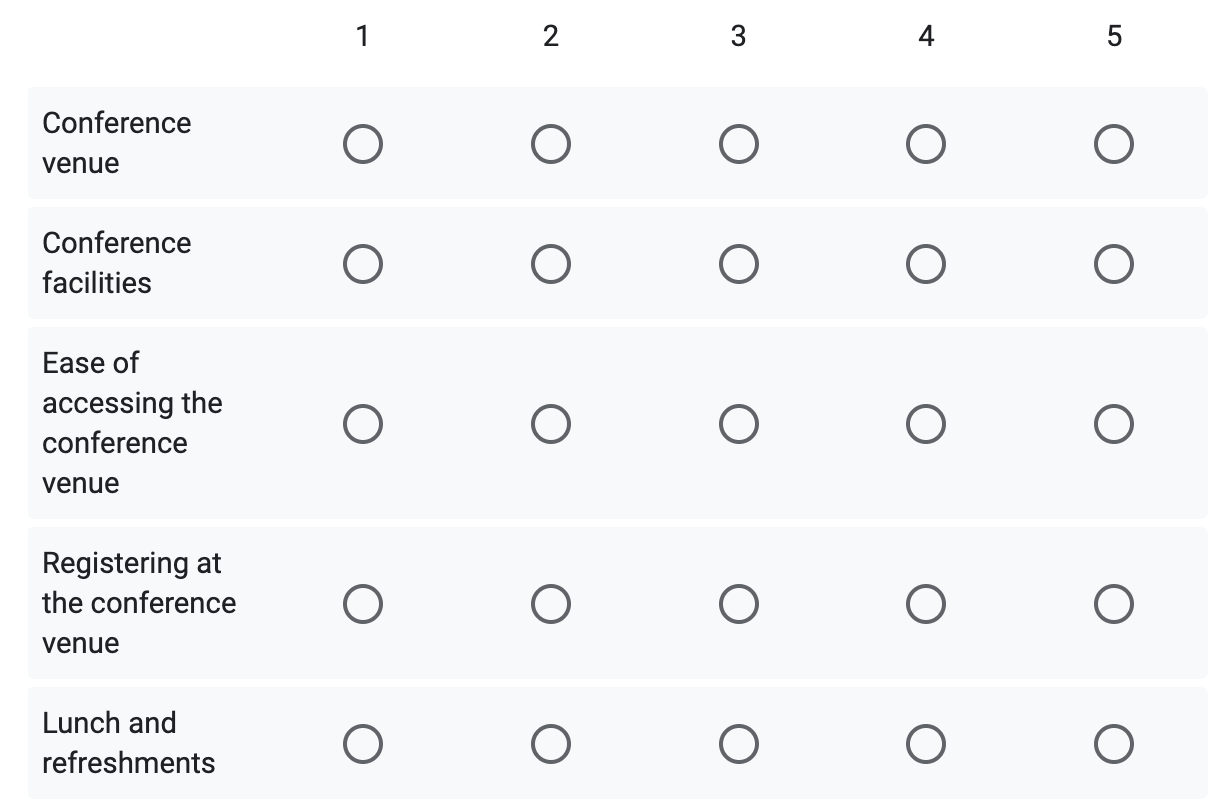


How would you rate the EPIC 2024 overall? *


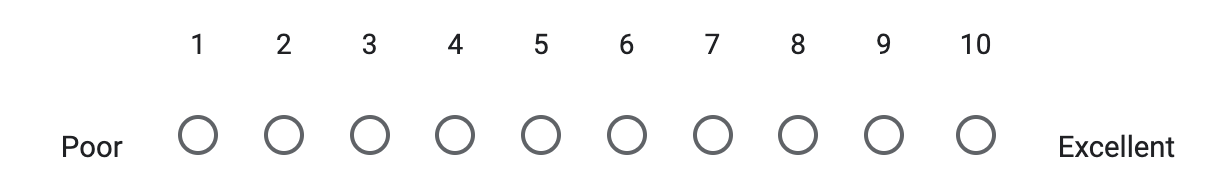


General Data Protection

We would like to save your contact information to inform you about future workshops and events. We respect your trust and protect your privacy, and therefore will never sell or share this data with any third parties outside of the University of Birmingham and our SIMBA team members. If uou have any questions, complaints or change your mind, contact [earlycareeredm@gmail.com](mailto:earlycareeredm@gmail.com). Please confirm below your consent for us to save your contact details. *

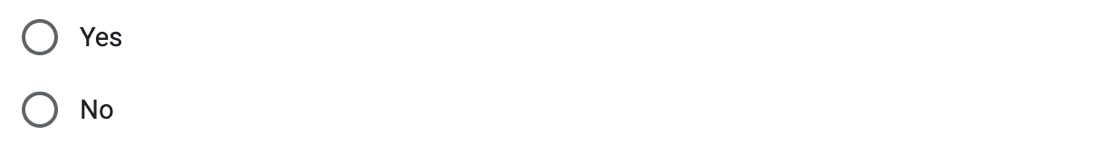


You have completed this evaluation. Thank you for your time!
