# Supplementary material for: Co-Designing and Evaluating a 1-Day Quality Improvement Workshop for Medical Students and Resident Physicians: Tutorial on Applying Kern’s Curriculum Development Framework
Source: JMIR Med Educ. 2026 Jun 17;12:e83657. doi: 10.2196/83657 (PMC13274911; doi:10.2196/83657)
Supplement: Multimedia Appendix 7 [file mededu-v12-e83657-s007.docx]

**Supplementary 8: Post-Workshop Interview**

Section 1: Introduction (5 mins)

1. Introduce the interviewer/facilitator
2. Thank participant for participating
3. Check participant’s audio and video are working
4. Confirm they are happy for us to record

START RECORDING

1. Introduce the interviewer/facilitator - Hi, I am Amanda, one of the facilitator for this research
2. Thank you for participating. Do you consent to the audio recording of your interview and using your anonymised quotes in research reports and publications?

Once they say yes, please continue with the below:

1. This interview is to understand medical students’ understanding of quality improvement projects and audits, and we would want to utilise this information to effectively address these specific needs and knowledge gaps so that future medical students can benefit from them.
2. There are no right or wrong answers. Your experiences and opinions are important and valid, and that is why we have invited you to speak with us today.
3. This interview will be kept confidential.
4. I have some questions I will ask to lead the conversation; otherwise, we want you to do the talking. If you think something is important, please bring it up, and if you don’t think a question or topic is relevant, please say so.
5. You can ask me to pause the recording at any time, and you are also free to leave the interview at any time.
6. Do you have any questions about the study itself or the interview?
7. If you want to find out more about how we handle your data, please refer to the participant information sheet that was emailed to you beforehand. If you have any questions about how we handle your data, please do ask me or get in contact with me via email.

Section 2: Conference Experience and Relevance

1. How was your overall experience at the conference, and how relevant was the content to you?
2. Did the conference meet your expectations? What parts were most valuable to you?
3. Do you think the workshops you experienced would have been beneficial to you before having to conduct the first ever audit?
4. Is this something you would like to be implemented in the medical school as part of your curriculum?

Section 3: Knowledge and Understanding

1. How has your understanding of audits and QIPs improved from attending the conference? What did you learn?
2. How confident are you now in distinguishing between audits, QIPs, and implementation research?

Section 4: Application of Knowledge

1. How do you plan to use the knowledge and skills gained from the conference in your future work? Can you give examples?
2. Which quality improvement tools or methods are you excited to use, and how will you use them?

Section 5: Challenges and Improvements

1. Can you share a challenging situation you feel better prepared to handle now? What improvements would you suggest for future conferences?

Section 6: Ethical Considerations and Feedback

1. What ethical issues are important in audits and QIPs and how did the conference help you consider these?
2. How did the conference help you handle feedback and criticism?

Finish Recording
