# Supplementary material for: Co-Designing and Evaluating a 1-Day Quality Improvement Workshop for Medical Students and Resident Physicians: Tutorial on Applying Kern’s Curriculum Development Framework
Source: JMIR Med Educ. 2026 Jun 17;12:e83657. doi: 10.2196/83657 (PMC13274911; doi:10.2196/83657)
Supplement: Multimedia Appendix 8 [file mededu-v12-e83657-s008.docx]

**Supplementary 9: Themes and codes developed from thematic analysis from post-workshop survey**

| Excerpt | Code | Theme |
| --- | --- | --- |
| This conference has helped very much | Positive feedback from conference/workshop | Factors that Influence the Current Level of Confidence in Participating in Audits or QIPs after Conference |
| Better understanding of what they entail |  |  |
| The lectures and workshops and all the information given at the conference today |  |  |
| The talks explaining the two and how they are done |  |  |
| Well explained at the epic conference today |  |  |
| Interesting to hear how to overcome barriers in conducting QIP |  |  |
| Experience |  |  |
| This conference, looking at example audits and presentations. Learning from guest speakers and x. Very useful slides and advice |  |  |
| Basic knowledge on audits |  |  |
| Knowledge gained from workshops |  |  |
| Knowing exactly what it is and how to go about doing it. How to gather data and analyse it especially. |  |  |
| This conference has improved my knowledge of audits and QIP and how to go about doing them |  |  |
| A lot of new info, need some time to review and consolidate the process |  |  |
| The amount of exposure and education I’ve received |  |  |
| The workshops and presentations today have definitely helped. |  |  |
| Seeing examples of others work, knowing the steps and the workshops where we practiced as a group have improved my confidence |  |  |
| Got good experience from today’s session |  |  |
| I have been equipped with knowledge on how to do a QIP/Audit something which i had no idea about before |  |  |
| Being able to find a supervisor who’ll be able to guide me |  |  |
| Creating a project ourselves |  |  |
| I now know what the aim is and how to approach it. |  |  |
| More confident in preparation |  |  |
| The conference today focused on how, which I found very useful. |  |  |
| Baseline teaching on the steps involved |  |  |
| Doing the workshops on conducting an audit or QIP really helped |  |  |
| From the workshops today I have been provided with a lot more knowledge of the foundation of how to go about conducting a QIP project and the different aspects that must be considered, as well as what makes a QIP different to an audit |  |  |
| Having had the workshop today and begun planning a theoretical audit |  |  |
| During the workshops and conference, the steps involved in each were made clear, and the clear definitions of the differences between audits and QIPs was also helpful |  |  |
| No official medical school teaching, having to seek out extra opportunities to learnt about QIP and audits | Lack of teaching |  |
| Not much experience yet | Lack of experience |  |
| Still feel like I need exposure to the process and the statistical side to it |  |  |
| Still need some more resources and preparation | Lack of resources | Reasons for Feeling Inadequacy in Starting or Contributing to Audits and QIPs after Conference |
| I know the structure and have completed one in the past. It would still be useful to connect with others who are interested in completing audits |  |  |
| Still need some more resources and preparation | Lack of experience |  |
| Today has given me a bit more confidence, but I still feel unsure in how to proceed with one |  |  |
| I have more information but still need to do some more research |  |  |
| Better equipped than before but would still need guidance |  |  |
| UoB med school do not place as much emphasis on research teaching | Lack of teaching |  |
| Need more teaching on it |  |  |
| I feel more confident than I did before this session but still need more sessions |  |  |
| Better understanding of how to undertake them | Positive feedback from conference |  |
| I feel like I would be able to contribute |  |  |
| Very thorough session |  |  |
| Broken down the steps well at the conference |  |  |
| Learnt important skills |  |  |
| Lots of teaching |  |  |
| the workshops have been particularly useful in allowing us to practice how to come up with an audit idea and how to make Gannt charts. |  |  |
| I gained from confidence from epic conference |  |  |
| I am more aware of what it entails and how to go about getting started now. |  |  |
| Today has given me a bit more confidence, but I still feel unsure in how to proceed with one |  |  |
| I have more information but still need to do some more research |  |  |
| The conference has prepared me really well. |  |  |
| I feel I understand the steps practically |  |  |
| Yes I think workshops were very useful |  |  |
| Again the same today’s session have been very helpful |  |  |
| I now understand the process and steps that are required in order to do a QIP. |  |  |
| I feel more confident than I did before this session but still need more sessions |  |  |
| I know the structure and have completed one in the past. It would still be useful to connect with others who are interested in completing audits. |  |  |
| Better equipped than before but would still need guidance |  |  |
| Think I could contribute as part of a group |  |  |
| I think knowing the SMART questions and what the aims are of the Audit or QIP. |  |  |
| Yes, it has given some finer information |  |  |
| Think I need to cement my understanding from today |  |  |
| I have been given good pointers and a step by step guide to conducting qips/audits. I was also able to form a project timeline using a Gantt chart with my group which helped me to better visual the process. |  |  |
| I definitely know more about conducting an audit or QIP but I think I’d only be confident in conducting one with the right team |  |  |
| I am now aware of what tasks are involved in conducting a QIP and feel that I would be able to contribute to one at least in part. |  |  |
| The discussions with doctors and senior medical students have made audits and QIPs seem much more accessible to me now |  |  |
| The steps involved in each were really made clear during the conference |  |  |
| Workshops | Workshop | Most Valuable Aspects of the Conference |
| Discussing concepts in the group project |  |  |
| Interactive discussion |  |  |
| Discussion with groups and advise from the speaker |  |  |
| Gantt workshop |  |  |
| Brainstorming ideas |  |  |
| Practicing brainstorming ideas and producing granny charts myself |  |  |
| Group work |  |  |
| Interactive nature |  |  |
| Creating the Gantt chart as it made you think about each individual step and makes you re-think aspects |  |  |
| Discussions and direct feedback |  |  |
| Going through it after we presented and hearing the feedback for all the groups |  |  |
| Planning the audit |  |  |
| Theini create your own QIP/Audit |  |  |
| Making our own Gantt chart and starting to write what we’d do for an audit |  |  |
| The interactive PCOS workshops. |  |  |
| Presenting information. And the presentations |  |  |
| the oral presentations and the workshops |  |  |
| The advice given and talks were really useful |  |  |
| It was good to see the different presentations and interactive sessions |  |  |
| Presentations and in particular the insight from the seniors. |  |  |
| Hands on activities |  |  |
| I really appreciated the workshops interleaved with the lectures and the way in which the whole day was organised in order of how a QIP would be conducted. It was really helpful to plan a QIP and be given some ideas of what QIPs have been done in the past and how impactful they have been |  |  |
| The introductory presentations and the example presentations of the audits and Gantt charts |  |  |
| The workshops and lectures |  |  |
| The lectures | Lectures |  |
| Discussion of difference between QIPs and Audits |  |  |
| The talk from the MD was good |  |  |
| The tips from x and the 4th years |  |  |
| The presentation discussing audits and qip |  |  |
| The presentations and speakers |  |  |
| I really appreciated the workshops interleaved with the lectures and the way in which the whole day was organised in order of how a QIP would be conducted. It was really helpful to plan a QIP and be given some ideas of what QIPs have been done in the past and how impactful they have been |  |  |
| The introductory presentations and the example presentations of the audits and Gantt charts |  |  |
| The workshops and lectures |  |  |
| The oral presentations and the workshops | Oral presentations |  |
| Maybe having fewer presentations | Oral presentations | Least valuable aspects of the workshop |
| Too many presentations |  |  |
| Audit presentations - they were useful to see examples but there were quite a lot to listen to. |  |  |
| Making the Gantt charts was less useful for a hypothetical audit | Gantt chart |  |
| Gantt chart was a bit too hypothetical, would be better to apply to a simple scenario e.g. baking a cake |  |  |
| Gantt charts were difficult to see on big screen | Technical issues |  |
| Time management/ some sessions running over | Practical issues |  |
| the first workshop could have had more guidance |  |  |
| A bit of repetition but probably good |  |  |
| Not sure as it was all valuable, perhaps coming up with a hypothesis for PCOS as there was limited time |  |  |
| Maybe the group discussions, but nothing was really not valuable | Group discussions |  |
| Will try and use the key concepts and buzz words | Active participation | Applications of knowledge obtained in workshop to medical education or future practice |
| Using frameworks and tips to define audit questions |  |  |
| I hope to be able to work on audits outside of med school |  |  |
| To actively participate in audits and QIPs |  |  |
| Useful for the audit in 4th year |  |  |
| Conduct more audits correctly |  |  |
| Lead QIPs for students |  |  |
| During future audits |  |  |
| Trying to network and get involved in audits or QIP. Trying to get more exposure and experience |  |  |
| Practicing |  |  |
| Y4 audits |  |  |
| Get more involved in future |  |  |
| By using the skills for my next year audit. |  |  |
| I will be able to apply what should be included in an audit for my 4th year audit |  |  |
| To the audit i need to do for SSE and 4th year |  |  |
| Use it to further my own QIP |  |  |
| Will apply the step-by-step approach |  |  |
| In my planning, organisation and carrying it out |  |  |
| Start a QIP |  |  |
| I have taken notes from the session which i will be using for my audit project next year |  |  |
| I’ll take forward what I’ve learnt into next year when I do my own audit |  |  |
| I hope to complete an audit in the coming months and will use the Gantt chart format for my own audit. |  |  |
| Creating a project for my elective |  |  |
| Will apply the structure i have learned that make up an audit |  |  |
| Will be taken this into fourth year. |  |  |
| In Audits and QIPs, hopefully lead by myself. |  |  |
| I will be revisiting this content and using the resources signposted to (hopefully) start my own audit this coming academic year |  |  |
| Take the information and use it when conducting an audit in year 4 |  |  |
| I intend on either planning an idea for a QIP or asking a supervisor if they have ideas so that I can partake in a QIP and gain experience of actually doing one |  |  |
| When we perform our compulsory audit in 4th year and any additional audits, I hope to be involved in |  |  |
| I plan on using the advice to talk to people and network to find future audit/QIP opportunities |  |  |
| No, please can we get the slides they were amazing! | Slide provision | Suggestions for improvement in future workshops |
| Yes. Possibly handouts and slides made available to help carry this knowledge throughout. |  |  |
| Making it slightly shorter | Time management |  |
| Gantt charts section could be trimmed down |  |  |
| More time to connect with others about audits and perhaps pairing clinicians with audit interests with students interested in conducting audits. |  |  |
| Maybe less audit presentations or split them by allowing a break in between. Or half in the morning, half in the afternoon. |  |  |
| More participation from other centres in the UK | Wider coverage |  |
| Sending this information to a wider audience |  |  |
| Can do similar sessions for junior doctors |  |  |
| Maybe less presentations or a break halfway through presentations as I lost my concentration :) | Oral presentations |  |
| More frequent sessions in year 3 | Frequency |  |
| More interactive workshops |  |  |
| Keep doing such workshops more frequently |  |  |
| Do more workshops! I’ll be there. |  |  |
| First workshop: maybe allocate specific questions for us to create a QIP on (ones that have NICE guidelines and guide our structure) | Guidance in workshops |  |
| More speakers | Speakers |  |
