# Supplementary material for: Co-Designing and Evaluating a 1-Day Quality Improvement Workshop for Medical Students and Resident Physicians: Tutorial on Applying Kern’s Curriculum Development Framework
Source: JMIR Med Educ. 2026 Jun 17;12:e83657. doi: 10.2196/83657 (PMC13274911; doi:10.2196/83657)
Supplement: Multimedia Appendix 9 [file mededu-v12-e83657-s009.docx]

**Supplementary 5: Themes and domains developed from thematic analysis from pre-workshop interview**

| Code | Theme | Domain (n) |
| --- | --- | --- |
| Administration | Challenges faced whilst doing QIP, audits, and methods to overcome them | Challenges and Solutions in QIPs and Audits (133) |
| Avoidance |  |  |
| Coming up with unique ideas |  |  |
| Confidence |  |  |
| Data collection |  |  |
| Funding |  |  |
| IT and data collection |  |  |
| Lack of incentives |  |  |
| Lack of motivation |  |  |
| Motivation |  |  |
| Non-standardization across data documentation |  |  |
| Prioritization |  |  |
| Resource intensity |  |  |
| Time and resources |  |  |
| Ask for help | Methods to address a challenging situation in a clinical setting |  |
| Boundary setting |  |  |
| Courageous |  |  |
| Reflection |  |  |
| Stay calm |  |  |
| Plan a strategy | Methods to handle constructive criticism before the workshops |  |
| Reflection |  |  |
| Take it as a challenge |  |  |
| Think of a bigger picture |  |  |
| Patient safety | Experience with positive impact of quality improvement in healthcare | Education and Skills Development in QIP and Audits (626) |
|  | Experience with teamwork and collaboration in a clinical setting |  |
|  | Failures of healthcare system in involving medical students to involve in QIP or audits |  |
|  | Ideas on the key principles of QI in healthcare |  |
| Changes to med curriculum | Importance of having an early exposure to QIP and audits for medical students |  |
|  | Importance of teamwork and collaboration in a clinical setting |  |
|  | Initial ease in collecting and analysing data |  |
| Importance of audits in healthcare | Initial understanding and appreciation of audits |  |
|  | Initial understanding on QIP in healthcare |  |
| Outcomes | Interviewees' roles and outcomes in previous QIP or audits before workshop |  |
| Roles |  |  |
| No | Participation in QIP or audits whilst in university |  |
| Yes |  |  |
| Improve patient care | Positive feedback on quality improvement project in healthcare |  |
| Increase patient safety and quality of care |  |  |
| Maximize healthcare outcomes |  |  |
| Provide targeted intervention |  |  |
| Workload reduction |  |  |
| 6 sigma | Previous experience with QI tools or methodologies |  |
| No experience |  |  |
| PDSA Cycle |  |  |
| Gain more experience | Reasons for attending the workshop on quality improvement and audits |  |
| Importance for career |  |  |
| Improved understanding |  |  |
| Learning from experienced doctors |  |  |
| Listen to experience |  |  |
| More understanding |  |  |
| Networking |  |  |
| Usefulness for future career |  |  |
|  | Recommendations for the workshop or conference |  |
|  | Reflection on the shortcomings of healthcare and potential approaches |  |
| Critical thinking | Skills possessed by interviewees to contribute in QIP and audit |  |
| Data collecting |  |  |
| Fast learner |  |  |
| Hardworking and dedicating |  |  |
| Inquisitively |  |  |
| Organization |  |  |
| Patience |  |  |
| Perseverance |  |  |
| Planning |  |  |
| Previous experience |  |  |
| Prioritization |  |  |
| Problem-solving |  |  |
| Team-working |  |  |
| Time management |  |  |
| Communication | Skills to improve teamwork and collaboration among healthcare professionals |  |
| Openness |  |  |
| Similar expectations |  |  |
| Audit loop closure | Specific areas of QI or audit to be learnt during workshop |  |
| Coming up with ideas |  |  |
| Data analysis |  |  |
| Data collection |  |  |
| Experience and practice |  |  |
| Intervention implementation |  |  |
| Conference | Suggested support or resources that would be beneficial for medical students |  |
| Data analysis software |  |  |
| Earlier introduction to QIPs |  |  |
| Online videos |  |  |
| Practical teaching like workshops |  |  |
| Technology |  |  |
| Compulsion | Suggestions for medical schools to better address students' needs and knowledge gaps in QIPs and audits |  |
| Detailed teaching |  |  |
| Early exposure |  |  |
| Practical examples |  |  |
| Practical teaching |  |  |
| Quality improvement week |  |  |
| Real-life example provision |  |  |
| Small group teaching |  |  |
| Support on practicalities |  |  |
| Views on medical schools’ contribution to the education on QIPs ad audits | Views on medical schools' contribution to the education on QIP and audits |  |
| Abide by trust confidentiality | Confidence in patient safety and patient confidentiality in QIP | Patient Safety, Confidentiality, and Ethical Considerations (110) |
| Avoid recognizable data |  |  |
| Data anonymization |  |  |
| Data generalization |  |  |
| Data separation |  |  |
| Ethical approval |  |  |
| Minimize data collection time points |  |  |
| Obtain ideas from experts |  |  |
| Retrospective data collection |  |  |
| Secure data storage |  |  |
| Transparency |  |  |
| Avoid biases | Ethical considerations in medical audits |  |
| Confidentiality |  |  |
| Data collection - not distressing |  |  |
| Honesty |  |  |
| Outcome consideration |  |  |
| patient confidentiality |  |  |
| Patient consent |  |  |
| Prior permission |  |  |
| Recommendation consideration |  |  |

Key: n = number of references
