# Supplementary material for: Co-Designing and Evaluating a 1-Day Quality Improvement Workshop for Medical Students and Resident Physicians: Tutorial on Applying Kern’s Curriculum Development Framework
Source: JMIR Med Educ. 2026 Jun 17;12:e83657. doi: 10.2196/83657 (PMC13274911; doi:10.2196/83657)
Supplement: Multimedia Appendix 10 [file mededu-v12-e83657-s010.docx]

**Supplementary 10: Themes and domains developed from thematic analysis from post-workshop interview**

| Code | Theme | Domain (n) |
| --- | --- | --- |
| Data anonymization | Ethical issues in QIPs and Audits | Ethical Considerations (111) |
| Data collection |  |  |
| Patient confidentiality |  |  |
| Patient consent |  |  |
| Prior permission |  |  |
| Recommendation consideration |  |  |
| Round Table Idea | Handling feedback and criticism |  |
| Supportive and constructive environment |  |  |
| Asking for ethical advice | Influence of Conference on the Consideration around Ethical Issues in QIPs and Audits |  |
| Ethical committees |  |  |
| Patient confidentiality |  |  |
| Current audit applications | Application of knowledge and skills gained from conference | Future Applications and Recommendations (191) |
| Future careers |  |  |
| Proactivity as a student |  |  |
|  | Preference over audits, QIPs, or implementation research |  |
| Advertisements | Suggestions for future conference |  |
| Delivery mode |  |  |
| Facilitators |  |  |
| Information provision and Signposting |  |  |
| Networking session |  |  |
| Pacing |  |  |
| Sitting arrangements |  |  |
| Timing and Frequency of Conference |  |  |
| Topic of discussions |  |  |
| Data analysis software | Useful Quality Improvement Tools or Methods |  |
| PDSA Cycle |  |  |
| Planning and Task Allocation |  |  |
| Technology |  |  |
| Time management and Gantt Chart |  |  |
|  | Usefulness in having this conference before conducting first audit |  |
|  | Usefulness of 'Practical tips when it comes to doing efficient audits and QIPs' |  |
|  | Benefits of workshops over lectures in medical education | Knowledge and Skills Acquisition (178) |
| Asking for help | Challenging situation that feels better prepared to handle post-conference |  |
| Data collection |  |  |
| Oral presentations |  |  |
| Personal initiatives |  |  |
| Team-working |  |  |
|  | Confidence in distinguishing between audits, QIPs, and implementation research |  |
| Advice from experts | Enhanced knowledge on QIPs and audits after attending the conference |  |
| Audit loop closure |  |  |
| Barriers and facilitators |  |  |
| Data analysis |  |  |
| Data collection |  |  |
| Examples of projects |  |  |
| Implementation |  |  |
| Implementation research |  |  |
| Importance |  |  |
| Objective generation |  |  |
| Pilot studies |  |  |
| Planning with time management |  |  |
| Steps of the audit cycle |  |  |
| Task allocation |  |  |
| Team-working |  |  |
| Use of technology |  |  |
| Overall Experience and Relevance of Conference | Expectations met | Positive Evaluation of the Workshop (338) |
| Positive feedback about the conference |  |  |
| Valuable section(s) of the conference |  |  |
| Importance of an MDT for research | Interactions between medical students of different levels and junior doctors |  |
|  | Introduction to artificial intelligence in medical education |  |
| Timing | Possibility for the implementation of similar workshop in medical curriculum |  |
| Use of CTFs in hospital academies |  |  |

Key: n = number of references
